# Mitochondrial fatty acid synthesis and MECR regulate CD4^+^ T cell function and oxidative metabolism

**DOI:** 10.1093/jimmun/vkaf034

**Published:** 2025-04-09

**Authors:** KayLee K Steiner, Arissa C Young, Andrew R Patterson, Ayaka Sugiura, McLane J Watson, Samuel E J Preston, Anton Zhelonkin, Erin Q Jennings, Channing Chi, Darren R Heintzman, Andrew P Pahnke, Yasmine T Toudji, Zaid Hatem, Matthew Z Madden, Emily N Arner, Allison E Sewell, Allison K Blount, Richmond Okparaugo, Emilia Fallman, Evan S Krystofiak, Ryan D Sheldon, Katherine N Gibson-Corley, Kelsey Voss, Sara M Nowinski, Russell G Jones, Denis A Mogilenko, Jeffrey C Rathmell

**Affiliations:** Division of Molecular Pathology, Department of Pathology, Microbiology and Immunology, Vanderbilt University Medical Center, Nashville, TN, United States; Department of Medicine, Vanderbilt University Medical Center, Nashville, TN, United States; Division of Molecular Pathology, Department of Pathology, Microbiology and Immunology, Vanderbilt University Medical Center, Nashville, TN, United States; Division of Molecular Pathology, Department of Pathology, Microbiology and Immunology, Vanderbilt University Medical Center, Nashville, TN, United States; Division of Comparative Medicine, Department of Pathology, Microbiology and Immunology, Vanderbilt University Medical Center, Nashville, TN, United States; Division of Comparative Medicine, Department of Pathology, Microbiology and Immunology, Vanderbilt University Medical Center, Nashville, TN, United States; Division of Rheumatology and Immunology, Department of Medicine, Vanderbilt University Medical Center, Nashville, TN, United States; Division of Molecular Pathology, Department of Pathology, Microbiology and Immunology, Vanderbilt University Medical Center, Nashville, TN, United States; Division of Molecular Pathology, Department of Pathology, Microbiology and Immunology, Vanderbilt University Medical Center, Nashville, TN, United States; Division of Molecular Pathology, Department of Pathology, Microbiology and Immunology, Vanderbilt University Medical Center, Nashville, TN, United States; Division of Rheumatology and Immunology, Department of Medicine, Vanderbilt University Medical Center, Nashville, TN, United States; Division of Molecular Pathology, Department of Pathology, Microbiology and Immunology, Vanderbilt University Medical Center, Nashville, TN, United States; Division of Molecular Pathology, Department of Pathology, Microbiology and Immunology, Vanderbilt University Medical Center, Nashville, TN, United States; Division of Molecular Pathology, Department of Pathology, Microbiology and Immunology, Vanderbilt University Medical Center, Nashville, TN, United States; Division of Molecular Pathology, Department of Pathology, Microbiology and Immunology, Vanderbilt University Medical Center, Nashville, TN, United States; Division of Molecular Pathology, Department of Pathology, Microbiology and Immunology, Vanderbilt University Medical Center, Nashville, TN, United States; Division of Molecular Pathology, Department of Pathology, Microbiology and Immunology, Vanderbilt University Medical Center, Nashville, TN, United States; Division of Molecular Pathology, Department of Pathology, Microbiology and Immunology, Vanderbilt University Medical Center, Nashville, TN, United States; Division of Rheumatology and Immunology, Department of Medicine, Vanderbilt University Medical Center, Nashville, TN, United States; Department of Cell and Developmental Biology, Vanderbilt University, Nashville, TN, United States; Mass Spectrometry Core, Van Andel Institute, Grand Rapids, MI, United States; Division of Comparative Medicine, Department of Pathology, Microbiology and Immunology, Vanderbilt University Medical Center, Nashville, TN, United States; Division of Molecular Pathology, Department of Pathology, Microbiology and Immunology, Vanderbilt University Medical Center, Nashville, TN, United States; Department of Metabolism and Nutritional Programming, Van Andel Institute, Grand Rapids, MI, United States; Department of Metabolism and Nutritional Programming, Van Andel Institute, Grand Rapids, MI, United States; Metabolism and Nutrition Program, Van Andel Institute, Grand Rapids, MI, United States; Division of Comparative Medicine, Department of Pathology, Microbiology and Immunology, Vanderbilt University Medical Center, Nashville, TN, United States; Vanderbilt Center for Immunobiology, Vanderbilt University Medical Center, Nashville, TN, United States; Division of Molecular Pathology, Department of Pathology, Microbiology and Immunology, Vanderbilt University Medical Center, Nashville, TN, United States; Vanderbilt Center for Immunobiology, Vanderbilt University Medical Center, Nashville, TN, United States

**Keywords:** CD4^+^ T cells, lipid metabolism, MECR, mtFAS

## Abstract

Imbalanced effector and regulatory CD4^+^ T cell subsets drive many inflammatory diseases. These T cell subsets rely on distinct metabolic programs, modulation of which differentially affects T cell fate and function. Lipid metabolism is fundamental yet remains poorly understood across CD4^+^ T cell subsets. Therefore, we performed targeted in vivo CRISPR/Cas9 screens to identify lipid metabolism genes and pathways essential for T cell functions. These screens established mitochondrial fatty acid synthesis genes *Mecr*, *Mcat*, and *Oxsm* as key metabolic regulators. Of these, the inborn error of metabolism gene *Mecr* was most dynamically regulated. *Mecr^fl/fl^*; *Cd4*^cre^ mice had normal naïve CD4^+^ and CD8^+^ T cell numbers, demonstrating that MECR is not essential in homeostatic conditions. However, effector and memory T cells were reduced in *Mecr* knockout and MECR-deficient CD4^+^ T cells and proliferated, differentiated, and survived less well than control T cells. Interestingly, T cells ultimately showed signs of mitochondrial stress and dysfunction in the absence of MECR. *Mecr*-deficient T cells also had decreased mitochondrial respiration, reduced tricarboxylic acid intermediates, and accumulated intracellular iron, which appeared to contribute to increased cell death and sensitivity to ferroptosis. Importantly, MECR-deficient T cells exhibited fitness disadvantages and were less effective at driving disease in an in vivo model of inflammatory bowel disease. Thus, MECR-mediated metabolism broadly supports CD4^+^ T cell proliferation and survival in vivo. These findings may also provide insight to the immunological state of MECR- and other mitochondrial fatty acid synthesis–deficient patients.

## Teaser

The Editors have selected this article as a highlight of the issue.

## Introduction

T cell metabolism is fundamentally integrated with cell differentiation and function, as each T cell subset requires a distinct metabolic program to reach its potential.^1^^,^^2^ T cell metabolism can therefore be targeted to affect the outcome of a variety of inflammatory diseases and cancer.^2^^,^^3^ Lipid metabolism controls a range of T cell biosynthetic, signaling, and energetic processes but differentially affects T cell subsets. T helper 1 (Th1), Th17 CD4^+^, and effector CD8^+^ T cells increase lipid synthesis upon activation, while T regulatory (Treg) cells rely on fatty acid β-oxidation.^1^^,^^4^^,^^5^ These pathways can also modulate T cell fate in inflammatory disease models. Inhibiting lipid metabolic protein ACC1 (acetyl-CoA carboxylase 1) in vivo dysregulates the formation of Th17 CD4 T cells, promotes Treg cell development, and prevents the Th17-mediated autoimmune disease experimental autoimmune encephalomyelitis.^6^^,^^7^ Conversely, the uptake of oxidized lipids can contribute to T cell dysfunction in tumors.^8^ However, the roles of specific lipid metabolic enzymes or processes in inflammatory diseases have not been explored through unbiased genetic screens.

Mitochondrial fatty acid synthesis (mtFAS) generates mitochondrial lipids independent of the cytosolic fatty acid synthesis (FAS) pathway. mtFAS utilizes acetyl-CoA and acyl carrier protein (ACP) to generate fatty acid chains 8 carbons in length or longer. Fatty acids in mtFAS do not contribute to triglycerides or phospholipids but instead generate octanoic acid for the synthesis of lipoic acid and long chain fatty acids.^9^ Lipoic acid is a cofactor for pyruvate dehydrogenase (PDH) and α-ketoglutarate dehydrogenase (OGDH). Because these enzymes have key roles for carbon entry into the tricarboxylic acid (TCA) cycle, dysfunction of the mtFAS pathway can broadly impair mitochondria.^10^ Longer acyl chains generated from mtFAS have been implicated in electron transport chain (ETC) stability and mitochondrial translation^11^ and proposed as indispensable to maintain mitochondrial respiration.^12^^,^^13^

Mitochondrial trans-2-enoyl coenzyme A reductase (MECR) catalyzes the last step in mtFAS to create acyl-ACP.^14^ MECR has also been associated with iron and ceramide metabolism, and MECR deficiency leads to an increase in both these pathways due to defective mitochondrial iron-sulfur (Fe-S) cluster biogenesis.^15^ Rare loss-of-function *MECR* mutations in humans were first described in 2016 with patients shown to have a mitochondrial deficiency disorder with dystonia and optic atrophy now termed mitochondrial enoyl CoA reductase protein–associated neurodegeneration (MEPAN) syndrome.^16^ However, the immune phenotypes in these patients have not been investigated in detail.

Here we employed unbiased approaches to identify essential lipid metabolic processes in CD4^+^ T cells. In vivo CRISPR/Cas9 screens performed using a custom lipid metabolism–based guide RNA library made from Twist Bioscience identified mtFAS as having a previously undescribed role in T cells. To further explore this pathway, we generated *Mecr*^fl/fl^; *Cd4*^cre^ mice. While resting T cells were not affected by *Mecr* deletion, activated MECR-deficient T cells proliferated poorly and had reduced overall mitochondrial and oxidative metabolism. Intracellular iron was also elevated consistent with increased susceptibility to ferroptosis. MECR deficiency decreased T cell fitness and led to reduced inflammation and disease severity in an inflammatory bowel disease (IBD) model of colitis. These findings show that although not essential for resting T cell viability, mtFAS and MECR promote fitness of activated T cells.

## Materials and methods

### Cell lines

PlatE cells (Cell Biolabs; RV-101) were used for transfection and retroviral production of single guide RNA (sgRNA). PlatE cells for passaging were cultured with 1 µg/mL puromycin (Gibco; A11138-03) and 10 µg/mL blasticidin (Gibco; A11139-03). MC38-OVA colon cancer tumor cells were generated as previously described.^17^ Cell lines were mycobacterium tested and fingerprinted.

### Mice

*Rag1^−/−^* (002216), Cas9 (024858), *Cd4*^cre^ (022071), OT-I (003831), and OT-II (004194) mice were obtained from the Jackson Laboratory. *Rag1^−/−^* mice were used for in vivo CRISPR/Cas9 adoptive transfer recipients and Cas9 mice were used for CRISPR/Cas9 experiments to isolate T cells that express Cas9. *Mecr*^fl^ mice were obtained from GenPharmatech (T007874) and crossed to *Cd4^cre^* mice to generate *Mecr^fl/fl^*; *Cd4^cre^*. Genotypes were validated with DNA sequencing through Transnetyx. For IBD models, 8- to 20-wk-old male and female *Rag1^−/−^* mice were used and euthanized when humane endpoint was reached (>20% weight loss). For injectable tumor models, 8- to 20-wk-old male and female *Rag1^−/−^* mice were used. Mice were euthanized when humane endpoint was reached (2 cm dimension, ulceration, weight loss >10%). Both male and female mice were utilized in experiments with Cre− and Cre+ cohoused and littermates. All mouse procedures were performed under Institutional Animal Care and Use Committee–approved protocols from Vanderbilt University Medical Center and conformed to all relevant regulatory standards.

### T cell isolation

Mouse spleens and lymph nodes were dissociated with a syringe, passed through a 70 μm filter, and lysed using ACK lysis buffer. CD4^+^ T cells were isolated using the StemCell EasySep Mouse CD4^+^ T cell Isolation Kit (StemCell Technologies; 19852) according to the manufacturer’s protocol. For CD4^+^ naïve cell isolation, the Naïve CD4^+^ T Cell Isolation Kit (Miltenyi Biotec; 130-104-453) was used according to the manufacturer’s protocol. Isolated cells were resuspended at 1 million cells/mL in complete RPMI 1640 (Corning; MT10040CV) media supplemented with 10% fetal bovine serum (FBS) (Avantor; 97068-085), 100 U/mL penicillin/streptomycin (Gibco; 15140122), 10 mM HEPES (Thermo Fisher Scientific; 15630080), 2 mM glutamine (Media Tek; 25030081), and 50 µM 2-mercaptoethanol (Gibco; 21985023) and cultured at 37 °C with 5% CO_2_.

### T cell activation and differentiation

Primary CD4^+^ T cells were activated using plate-bound anti-CD3 (3 mg/mL) (Invitrogen; 16-0031-86) and anti-CD28 (2 mg/mL) (Invitrogen; 16-0281-86) antibodies at 1 million cells/well in a tissue culture–treated 24-well plate (Thermo Fisher Scientific; 142475). A total of 10 ng/mL recombinant human interleukin (IL)-2 (Frederick National Laboratory; RO 23-6019) was added to bulk CD4 T cell cultures. Naïve T cells were skewed to the following subsets with cytokines: Th1 (10 ng/mL mouse IL-12 [mIL-12] [BioLegend; 577004], 10 ng/mL recombinant human IL-2, 1 µg/mL anti-IFNγ [Bio X Cell, BP0055], 10 µg/mL anti-IL-4 [Bio X Cell, BP0045], Th2 (10 ng/mL recombinant human IL-2, 80 ng/mL mIL-4 [PeproTech; 214-14], 10 µg/mL anti-interferon γ (anti-IFNγ), 10 µg/mL anti-IL-12 [Invitrogen; 16-7123-85]), Th17 (50 ng/mL mIL-6 [Miltenyi Biotec; 130-096-685], 10 ng/mL mIL-23 [Miltenyi Biotec; 130-096-677], 10 ng/mL mIL1β [Miltenyi Biotec; 130-094-053], 1 ng/mL human transforming growth factor β1 [TGF-β1] [PeproTech; 100-21], 10 µg/mL anti-IFNγ, 10 µg/mL anti-IL-4]), iTreg (10 ng/mL recombinant human IL-2, 1.5 ng/mL human TGF-β1, 10 µg/mL anti-IFNγ, 10 µg/mL anti-IL-4).

### Isolation of cells from the lamina propria and intraepithelial lymphocytes

Colons from the IBD model were washed 3 times with phosphate-buffered saline (PBS) and chopped into small pieces. Pieces were incubated in predigestion solution of Hanks’ balanced salt solution supplemented with 20 mM HEPES and 5 mM EDTA shaking at 37 °C. After, cells and solution were placed over a 100 µm filter into a conical tube. Colon fragments not passed through the filter were again incubated in predigestion solution shaking at 37 °C. This process was repeated 2 more times and cells passed through the filter represent the intraepithelial lymphocytes. After, pieces were digested with DNAse 1 and collagenase T3 using a gentleMACS dissociator and represents the lamina propria cells.

### Isolation and enrichment of cells from the liver and skin for flow cytometry

Euthanized mice were immediately perfused through the right ventricle with 30 mL of ice-cold PBS draining through the portal vein. The liver and ears were dissected, washed in PBS, minced to small pieces with scissors and digested for 30 min at 37 °C in Dulbecco’s Modified Eagle Medium + 0.5% bovine serum albumin (BSA) + 0.1% collagenase D. Cell suspension was filtered through a 70 μm strainer followed by centrifugation at 400 *g* for 10 min. Total cell pellets were resuspended in 2 mL 30% Percoll gradient and loaded on top of 1 mL 80% Percoll solution in PBS and centrifugated at 400 *g* for 20 min. The cells that accumulated in the interface between 30% and 80% Percoll solutions (the fraction enriched with immune nonparenchymal cells) were carefully collected and washed with ice-cold PBS containing 0.5% BSA by centrifugation at 400 *g* for 10 min. The cell pellet was resuspended in ice-cold PBS containing 0.5% BSA stained with antibodies for flow cytometry analysis.

### CRISPR screen and sgRNA generation

The targeted lipid metabolism library was generated by referencing the KEGG Pathway Database (https://www.genome.jp/kegg/pathway.html) in addition to genes found in literature searches. Four sgRNAs per each gene found in the CRISPR Knockout Pooled Library (Brie) along with 10 nontargeting controls (NTCs) for a total of 47 genes and 198 targets. The sgRNA sequences flanked by the following adaptor sequences were purchased as an oligo pool from Twist Bioscience: GGAAAGGACGAAACACCGXXXXXXXXXXXXXXXXXXXXGTTTTAGAGCTAGAAATAGCAAGTTAAAATAAGGC. The library was then prepared according to Sugiura et al.^18^ Briefly, the vector was cloned into a pMx-U6-gRNA-BFP–expressing retroviral packaging vector and the resulting plasmid library was amplified and electroporated into ElectroMAX DH10B *E. coli* (Invitrogen; 18290015) to maintain >50-fold coverage.

DNA was transfected into Plat-E retroviral packaging cell line using Polyplus jetOPTIMUS DNA and siRNA transfection reagent (Polyplus; 101000051). Dulbecco’s Modified Eagle Medium media (without puromycin and blasticidin) was changed 4 h post-transfection, and viral supernatant produced by the transfected PlatE cells was collected after an additional 48 h of culture.

### IBD model CRISPR screen

Naïve CD4^+^ T cells were isolated from the spleens and lymph nodes from Cas9-transgenic mice and were activated with RPMI Th17 skewing media and cytokines. 48 h post–T cell activation, the BFP-expressing CRISPR library viral supernatant was spun onto retronectin (Takara Bio; T100B) coated non–tissue culture plates at 2,000 *g* for 2 h at 32 °C. Activated T cells were transferred to the plates and spun for another 15 min at 2,000 *g* at 32 °C, then put in the incubator at 37 °C, 5% CO_2_. One day post–T cell transduction, a sample of the cells were collected at 1,000-fold representation of the library. Additionally, 4 million live BFP^+^ transduced T cells were adoptively transferred into each *Rag1^−/−^* mice by intraperitoneal (i.p.) injection. Mice were weighed twice weekly until 20% weight loss of after 8 wk. CD4^+^ T cells from the mesenteric lymph nodes and lamina propria were isolated and T cells were isolated using a positive selection using CD4 microbead kit (Miltenyi Biotec; L3T4).

### OT-II lung inflammation model CRISPR screen

CD4^+^ T cells were isolated from the spleen and lymph nodes of OT-II; Cas9 double-transgenic mice and activated with splenocytes irradiated at 30 Gy and OVA peptide (10 mg/mL) (Sigma Aldrich; O1641-5mg) for OT-II lung inflammation model. A total of 48 h post–T cell activation, the BFP-expressing CRISPR library viral supernatant was spun onto retronectin (Takara Bio; T100B) coated non–tissue culture plates at 2,000 *g* for 2 h at 32 °C. Activated T cells were transferred to the plates and spun for another 15 min at 2,000 x g at 32 °C, then put in the incubator at 37 °C, 5% CO_2_. One day post–T cell transduction, a sample of the cells were collected at 1,000-fold representation of the library. Additionally, 3 million live BFP^+^ transduced T cells were adoptively transferred into each *Rag1^−/−^* mice by i.p. injection. On days 1, 3, 5, and 7 post–adoptive transfer, mice were sensitized with intranasal ovalbumin protein, resulting in CD4^+^ T cells infiltrating the lung. On day 8 post–adoptive transfer, lungs were collected and digested with Type 4 collagenase and DNase and dissociated with a GentleMACS dissociator under m_lung_02.01 protocol. Digestion was stopped by adding 0.5M EDTA and digested tissue was filtered through a 70 µM filter, centrifuged to pellet cells, and T cells were isolated by positive selection using CD4 microbead kit (L3T4).

### MC-38 OVA adoptive transfer tumor model CRISPR screen

*Rag1^−/−^* were injected subcutaneously with 1 million MC38-OVA tumor cells. After 16 d, CD8^+^ T cells were isolated from the spleen of an Cas9; OT-I mouse using a negative isolation kit (Miltenyi Biotec; 130-095-236) according to the manufacturer’s protocol and activated for 2 d, then transduced with the BFP-expressing lipid metabolism CRISPR library. Mice were injected with 10 million transduced CD8^+^ Cas9; OT-1 T cells retro-orbitally. Mice were taken down at humane endpoint or after 7 d. Tumors were chopped and mechanically dissociated on a the Miltenyi Biotec gentleMACS Octo Dissociator with Heaters (setting implant tumor one) and digested with 435 U/mL DNase I (Sigma-Aldrich; D5025) and 218 U/mL collagenase (Sigma-Aldrich; C2674) at 37 °C for 30 min. Tumors were then passed through a 70 μm filter and lysed using ACK lysis buffer. CD8^+^ tumor-infiltrating lymphocytes were isolated using a positive selection kit (Miltenyi Biotec; 130 116 478) according to the manufacturer’s protocol and CRISPR library preparation, and sequencing was followed as previous.

### CRISPR screen preparation and sequencing

CRISPR Screen DNA isolation and processing was performed according to Sugiura et.al..^18^ Briefly, genomic DNA from cells was extracted (Millipore Sigma, EXPEXTKB) and sgRNA sequences were amplified by 2 rounds of polymerase chain reaction. Two technical replicates were analyzed with the first amplification round attaching adaptor primers and the second amplification attaching barcoded Illumina sequencing primers. The 2 amplicons were then purified by gel extraction, combined at equimolar, and sequenced for 150 cycles in paired-end mode on the Illumina Novaseq6000 platform at the Vanderbilt Technologies for Advanced Genomics (VANTAGE) core. At least 1,000-fold representation of the library was maintained throughout the process. Fastq files were analyzed using the Model-based Analysis of Genome-wide CRISPR/Cas9 Knockout (MAGeCKv0.5.0.3) method^19^ to determine statistical significance. The sgRNA frequencies in the CD4^+^ T cells isolated from the diseased tissue was compared with the sgRNA frequency in the CD4^+^ T cells at the time of adoptive transfer (day 0). Library and screening results are available online at (https://functionalimmunogenomics.shinyapps.io/crispr/).

### CRISPR/Cas9 single guide knockout

Single sgRNA knockouts were performed as above with the exception that sgRNAs specific to *Mecr* cloned into a BFP-expressing vector or NTC cloned into a GFP-expressing vector were used instead of the CRISPR library utilizing the following sequences of sg*Mecr* oligo cloned into pMx-U6-gRNA-BFP: *Mecr* F CACCGCGTGGCGGTACCAAGCCTCG *Mecr* R AAACCCAGGCTTGGTACCGCCACGC. Five days post-transduction, cells achieve knockout and are used for subsequent experiments.

### IBD adoptive transfer CRISPR/Cas9 1:1

Donor cells were prepared by isolating naïve CD4^+^ T cells (CD4^+^ CD44^−^) by magnetic bead isolation (Miltenyi Biotec; 130-104-453) from Cas9 transgenic mice. Cells were polarized to Th17 subset and cultured for 2 d. Cas9-expressing Th17 CD4^+^ T cells were transduced with the PlatE retroviral supernatant containing CRISPR sgRNA for *Mecr* single-guide knockout using a BFP-expressing vector or NTC in GFP-expressing vector and cultured for 2 d. 4 million total live cells at an equal amount of transduced T cells found by flow cytometry were transferred into *Rag1^−/−^*mice by i.p. injection. Mice were weighed twice weekly to monitor disease progression. Mice were sacrificed when mice lost 20% of their body weight or after 8 wk and spleen, mesenteric lymph nodes (mLNs), lamina propria, intraepithelial lymphocytes, and skin were collected for T cell phenotyping by gating on the transduced T cells of GFP (NTC) or BFP (*Mecr*-KO)

### IBD adoptive transfer Mecr^fl/fl^; CD4^cre^ mice

Donor cells were prepared by isolating naïve CD4^+^ T cells (CD4^+^ CD44^−^) by magnetic bead isolation (Miltenyi Biotec; 130-104-453) from *Mecr^fl/fl^*; *Cd4^cre^* – (WT) or + (*Mecr*-KO) mice. 400,000 live naïve CD4^+^ WT or *Mecr*-KO T cells were transferred into *Rag1^−/−^*mice by i.p. injection. Mice were weighed twice weekly to monitor disease progression. Mice were sacrificed when mice lost 20% of their body weight or after 8 wk and spleen and mesenteric lymph nodes were collected for T cell phenotyping. Colons were rolled for cassetting and fixed in 10% neutral buffered formalin for 3 d at room temperature before routine processing, paraffin embedding, sectioning at 4 µm, and hematoxylin and eosin staining. Colonic pathology and inflammation were scored in a semi-quantifiable manner by a board-certified veterinary pathologist based on Izcue et al.^20^ with proximal, middle, distal, and total colon histopathology scores as compared with PBS control. Histopathologic changes scored included inflammation both in the lamina propria and transmurally (0 = none; 1 = mild, rare, scattered; 2 = moderate, multifocal, 3 = moderate to severe, coalescing to diffuse), as well as the percentage of area affected in the slide (0 = <5%; 1 = 6%–30%; 2 = 31%–60%; 3 = >60%) and epithelial changes (epithelial cell hyperplasia, goblet cell depletion, erosions and/or ulcerations (0 = none; 1 = mild, rare, scattered; 2 = moderate, multifocal, 3 = moderate to severe, coalescing to diffuse).

### Flow cytometry

Single-cell suspensions were incubated in Fc block (1:50; BD; 553142) in 50 µL for 10 min at room temperature, stained for surface markers in 50 µL of fluorescence-activated cell sorting (FACS) buffer (PBS +2% FBS) for 15 min at room temperature, washed once with FACS buffer, and resuspended in 200 µL FACS buffer for analysis on a Miltenyi MACSQuant Analyzer 10 or 16. The eBioscience Foxp3/transcription factor staining buffer kit (Thermo Fisher Scientific; 00-5523-00) was used for intracellular staining of transcription factors. For intracellular cytokine staining, single-cell suspensions were incubated for 4 h at 37 °C and 5% CO2 in supplemented RPMI 1640 media supplemented with PMA (50 ng/mL; Sigma-Aldrich; P8139-1MG), ionomycin (750 ng/mL; Sigma-Aldrich; I0634-1MG), and GolgiPlug (1:1000, BD Biosciences, 555029) and processed using the BD Cytofix/Cytoperm Fixation and Permeabilization Solution (Thermo Fisher Scientific; BDB554722). Surface staining was performed as described previously. Afterward, cells were fixated and permeabilized for 20 min at 4 °C using 50 µL of the Cytofixation buffer, and then stained for intracellular markers in 50 µL diluted Cytoperm Permeabilization Solution for at least 30 min at 4 °C. For flow cytometry on BFP and GFP expressing cells with intracellular transcription factors, cells were fixed in 2% PFA for 20 min at room temperature before using the FoxP3/transcription factor kit to preserve BFP/GFP signal. Ghost Dye Red 780 viability dye (1:4000, Cell Signaling, 18452S) was used identically to surface antibodies. The antibodies used were CD4 BV510 (1:200; BD Biosciences; 563106), CD4 SB600 (1:200; Invitrogen; 2442209), CD4 e450 (1:200; Invitrogen; 48-0041-82), CD8a BV510 (1:200 BD Biosciences, 563068), CD8a APC (1:200; BioLegend; 100712), CD62L PE (1: 200; BioLegend; 104408), CD62L APC (1:200; BioLegend; 104412), CD44 PE-Cy7 (1:300; BioLegend; 103030), PD1 PE (1:200; eBioscience; 12-9985-83), CD71 PE-Cy7 (1:200; BioLegend; 113812), CD25 PE-Cy5 (1:100; Invitrogen; 15-051-82), CD45 FITC (1:200; BioLegend; 157214), CD127 PECy7 (1:50; BioLegend; 135014), KLRG1 APC (1:100; Invitrogen; 17-5893-82), Tbet PE-Cy7 (1:100; BioLegend; 64484), RORγt APC (1:100; Invitrogen; 17-6988-82), FoxP3 PE-Cy5 (1:100; Invitrogen; 15-5773-82), Gata3 PE (1:100; Invitrogen; 1-9966-62), TCF1 AF647 (1:100; Cell Signaling Technology; C63D9), Eomes PE (1:100; Invitrogen; 12-4875-82), Annexin V APC (1:100; BioLegend; 64090), IFNγ APC (1:100 BD Biosciences, 554413), IL-2 PE-Cy5 (1:100; BioLegend; 50384), IL-4 PE-Cy7 (1:100; BioLegend; 504118), IL-17a PE (1:100; Invitrogen; 12-7177-81), TNFα PE-Cy7 (1:100; BioLegend; 506324). P-S6 PE (1:100; Cell Signaling Technology; 5316S), Propidium Iodide (Sigma-Aldrich; P4864-10mL), BioTracker Far Red Labile 2+ (Millipore; SCT037), Mito-FerroGreen (Dojindo; M489-10). Cell Trace Violet (CTV) (Thermo Fisher Scientific; C34557) was used at 1:1,000 for cell proliferation assays. Mitochondrial mass was measured with 200 nM MitoTracker Green FM (Invitrogen; M7514), mitochondrial membrane potential was measured with 150 nM TMRE (Lifetech; T-669), mitochondrial reactive oxygen species (ROS) was measured with MitoSOX (Invitrogen; M36008), ROS was measured with CM-H2DCFDA (Lifetech, C6827), and C11 BODIPY (Thermo Fisher Scientific; D3861) was used to measure oxidized lipids. Each staining was incubated for 30 min at 37 °C 5% CO_2_ in PBS. Flow cytometry data were analyzed using FlowJo v10.7.1 (TreeStar). Gating strategies were drawn from singlets, lymphocytes, live cells, and CD4^+^ or CD8^+^ T cells.

### ELISA

Production of IFNγ and IL-10 from T cell culture supernatant was measured using Legend Max ELISA Kit Mouse IFNγ (BioLegend; 430807) and Mouse IL-10 (BioLegend; 431417) according to manufacturer’s protocol. Absorbance was read at 450 nm and 570 nm and values were normalized to live cell number determined by trypan blue counting.

### Western blot

Cells were lysed in Pierce RIPA Buffer (Thermo Fisher Scientific; 89900) with 1:100 Protease and Phosphatase Inhibitor Cocktail (Thermo Fisher Scientific; 78442) for 30 min on ice. Lysates were centrifuged for at 13,000 RPM 15 min at 4 °C to recover supernatant and quantified for protein concentration by BCA assay (Thermo Fisher Scientific; 23225). A total of 70 µg of protein was loaded per well for polyacrylamide gel electrophoresis using Mini-PROTEAN Precast Polyacrylamide Gels (Bio-Rad; 4561095). Gel was transferred to a low fluorescence PVDF membrane (Cytiva; 10600023) activated in methanol using 1X Tris-Glycine Transfer Buffer (KD Medical; RGF-3395, with 20% methanol). Membranes were blocked for 1 h with 5% milk with Tris-buffered saline + 0.5% Tween, before incubation with primary antibody overnight at 4 °C. Blots were incubated for 1 h at room temperature with anti-HRP antibodies and were visualized using Chemiluminescence Pico Plus (Thermo Fisher Scientific; 34580) via an Amersham Imager 600. The antibodies used for Westerns were: β-actin (1:4,000; Cell Signaling Technology; 3700), Vinculin (1:10,000; Abcam; ab129002), MECR (1:500; Thermo Fisher Scientific; 14932-1-AP), oxidative phosphorylation (OXPHOS) cocktail (1:250; Abcam; ab110413), lipoic acid (1:1,000 Millipore, 437695), anti-mouse IgG HRP (1:20,000; Promega; W402B), and anti-rabbit IgG HRP (1:20,000; Promega; W401B). Western blot quantification was done by using ImageStudioLite.

### Extracellular flux assay

T cells were plated at 150,000 live cells/well in 8 technical replicates on a Cell-Tak–coated plate (Corning; 354240) in Agilent Seahorse RPMI 1640 (Agilent; 103576-100) supplemented with 10 mM glucose (Agilent; 103577-100), 1 mM sodium pyruvate (Agilent; 103578-100), and 2 mM glutamine. Cells were analyzed on a Seahorse XFe 96 bioanalyzer using the Mitostress assay (Agilent; 103015-100) with 1 μM oligomycin (Cayman Chemical; 11342), 2 μM FCCP (Cayman Chemical; 15218), and 0.5 μM rotenone (Cayman Chemical; 13995)/antimycin A (Cayman Chemical; 19433). Data were then analyzed in the Agilent Wave software version 2.6.

### Transmission electron microscopy

All electron microscopy reagents were purchased from Electron Microscopy Sciences. Wild-type (WT) or *Mecr*-KO CD4^+^ T cells were activated for 3 d and cells were fixed with 2.5% glutaraldehyde in 0.1 M Na cacodylate buffer for 1 h at room temperature followed by 24 h at 4 °C. After fixation, the cells were sequentially postfixed with 1% tannic acid, 1% osmium tetroxide, and stained en bloc with 1% uranyl acetate for 1 h each. Samples were dehydrated in a graded ethanol series of 25%, 50%, 70%, 80%, 85%, 90%, and 100% (3 times) steps for 15 min each. Following dehydration, the samples were infiltrated with Quetol 651–based Spurr’s resin composed of Quetol 651, ERL 4221, NSA, DER 736, and BDMA (Electron Microscopy Sciences) at 50%, 75%, and 100% (3 times) resin exchanges using propylene oxide as the transition solvent. Samples were polymerized in Eppendorf tubes at 60 °C for 48 h. Ultrathin sections were prepared on a Leica UC7 ultramicrotome at a nominal thickness of 70 nm and collected onto 300-mesh nickel grids. Sections were stained with 2% uranyl acetate and lead citrate. Cell samples were imaged using a JEOL 2100+ operating at 200 kV equipped with an AMT NanoSprint CMOS camera using AMT imaging software.

### ^13^C Glucose tracing metabolomics

#### Preparation of T cells for stable isotope tracing

*Mecr^fl/fl^*; *Cd4^cre^*– (WT) or *Mecr*^fl/fl^; *Cd4*^cre^+ (*Mecr* knockout [*Mecr*-KO]) mice CD4^+^ T cells were purified from spleen and peripheral lymph nodes by using a CD4^+^ T cell negative selection kit following the manufacturer protocol (StemCell Technologies). Purified CD4^+^ T cells were activated and expanded for 72 h in Iscove’s Modified Dulbecco’s Medium supplemented with 10% dialyzed FBS (Wisent), L-glutamine (Invitrogen), penicillin-streptomycin (Invitrogen), 2-ME (Sigma-Aldrich), using plate-bound anti-CD3 (2 µg/mL; Thermo Fisher Scientific; 16-0031-86), anti-CD28 (1 µg/mL; Thermo Fisher Scientific; 16-0281-86), and IL-2 (50 U/mL; PeproTech; 212-12). After 72 h, the expanded WT and *Mecr*-KO CD4^+^ T cells were transferred into Van Andel Institute Media media^21^ containing 5 mM [U-^13^C_6_] glucose (Cambridge Isotope Laboratories) for 4 h at 37 °C. Immediately following incubation, cells were transferred from tissue culture plates to microfuge tubes and centrifuged at 600 *g* and 4 °C for 1 min. The cell pellet was washed 1 time with ice-cold saline and centrifuged before being snap frozen on dry ice and stored at −80 °C.

#### Metabolite extraction

Metabolites were extracted from frozen cell pellets in ice-cold acetonitrile:methanol:water (40%:40%:20% v/v) containing 1 µg/mL of D5 glutamate (DLM-556; Cambridge Isotope Laboratories) as an internal standard at a concentration of 2 × 10^6^ cells/mL of extraction solvent.^22^ Crude extracts were vortexed for 30 s, sonicated in a water bath sonicator for 5 min, and incubated on wet ice for 1 h. Extracts were precipitated by centrifugation at 14,000 *g* for 10 min at 4 °C, and the 800 µL of the soluble supernatant (1.6 × 10^6^ cell equivalents), were dried in a vacuum evaporator. Dried extracts were resuspended in 50µL of LCMS grade water, resulting in a final concentration of 3.2 × 10^7^ cell equivalents/mL.

#### LC-MS metabolomics

Metabolites were detected using a tributylamine ion-paired reversed phase chromatography coupled to an Orbitrap Exploris 240 mass spectrometer (Thermo Fisher Scientific).^22^ The column was a ZORBAX Rapid Resolution HD (2.1 × 150 mm, 1.8 µm; 759700–902; Agilent). Mobile phase A was 3% methanol, mobile phase B was 100% methanol, and each contained 10 mM tributylamine (90780; Sigma-Aldrich), 15 mM acetic acid and 2.5 µM medronic acid (5191–4506; Agilent Technologies). The LC gradient was 0–2.5 min, 0% B; 2.5–7.5 min, ramp to 20% B; 7.5–13 min, ramp to 45% B; 13–20 min, ramp to 99% B; 20–24 min, hold at 99% B. The flow rate was 0.25 mL/min, and the column compartment was heated to 35 °C. The column was then backflushed with 100% acetonitrile for 4 min (ramp from 0.25 to 0.8 mL/min in 1.5 min) and re-equilibrated with mobile phase A for 5 min at 0.4 mL/min. The H-ESI source was operated at spray voltage of −2,500 V, sheath gas of 60 arbitrary units (a.u.), aux gas of 19 a.u., sweep gas of 1 a.u., ion transfer tube at 300 °C, and vaporizer at 250 °C. Full scan MS1 data was collected from 70 to 800 m/z at mass resolution of 240,000 FWHM with RF lens at 35%, and standard automatic gain control. ddMS2 data were collected on unlabeled control samples using HCD fragmentation HCD at stepped collision energies of 20%, 35%, and 50%, with orbitrap detection at 15,000 full width at half maximum. Peak picking and integration was completed in Skyline (v23.3) using an in-house compound data base curated from analytical standards.^22^ Natural abundance correction was performed using IsocorrectoR.^23^

### Ferroptosis rescue

CD4^+^ T cells from *Mecr^fl/fl^*; *Cd4^cre^* mice were isolated and activated with anti-CD3, anti-CD28, and rhIL-2 in complete RPMI 1640 media and cultured for 3 d. Cells were then washed in PBS and resuspended at 1 × 10^6^ cells/mL in fresh media and pretreated for 2 h with 50 nM UAMC-3203 (Cayman Chemical; 26525) to specifically prevent ferroptosis. 0.5 µM (1S, 3R)-RSL3 (Cayman Chemical; 19288) or dimethyl sulfoxide solvent control was then applied overnight to induce ferroptosis. Viability and markers of ferroptosis were quantified the next day by flow cytometry.

### Deferoxamine rescue

CD4^+^ T cells from *Mecr*^fl/fl^; *Cd4*^cre^ mice were isolated and activated with anti-CD3, anti-CD28, and rhIL-2 in complete RPMI 1640 media. Cells were cultured for 3 d in the presence or absence of iron chelator deferoxamine (DFO) at 100 µM spiked into media after 4 h postactivation. After 3 d, viability, cell death, and functional markers were quantified by flow cytometry.

### RNA sequencing of isolated CD4^+^ T cells from in vivo IBD model

Following IBD disease development using transfer of naïve CD4^+^ T cells from WT or *Mecr*-KO mice into *Rag1^−/−^* mice, CD4^+^ T cells were isolated from the spleens of *Rag1^−/−^* mice using StemCell EasySep Mouse CD4^+^ T cell Isolation Kit (19852) according to the manufacturer’s protocol. RNA was isolated using RNeasy Plus Mini Kit (Qiagen; 74134) according to the manufacturer’s protocol and given to VANTAGE core at Vanderbilt University Medical Center for library preparation. Messenger RNA enrichment and complementary DNA library were prepared utilizing the stranded messenger RNA (polyA-selected) library preparation kit before sequencing on the Illumina NovaSeq X Plus.

#### Reads processing, quality control, and alignment

Paired-end fastq files for each sample were aligned to mouse genome GRCm39 RefSeq assembly GCF_000001635.27 (Release Feb, 2024). We used STAR^24^ in twopassMode to align the reads to the reference genome and sort them by coordinate. Each sample was evaluated according to a variety of both pre- and postalignment quality control measures with FastQC^25^ 0.12.1, Picard Toolkit 3.2.0. from the Broad Institute, RSeQC^26^ 5.0.4, MultiQC^27^ 1.25. Reads were trimmed with Trimmomatic^28^ 0.39. Polymerase chain reaction duplicates were marked with Picard tools, and the deduplication effect was further tested downstream. Aligned read counts were calculated by featureCounts^29^ from the subread 2.0.2 release. The pipeline for data processing is available at GitHub Repository (https://github.com/MogilenkoLabVUMC/RNAseq_pipelineDock_MECR_KayLee).

#### Transcriptomics analysis

Bulk RNA sequencing count data were then further analyzed in R 4.4.1 (R Foundation for Statistical Computing) and RStudio 2024.9.0.375. Differential expression analysis was performed using the edgeR^30^ 4.2.1 and limma-voom^31^ 3.60.6. Picard deduplication did not strongly affect the gene rankings (Spearman r = 0.991), but it introduced ties in the preranked genes list statistics during gene set enrichment analysis (52.54%). Un-deduplicated read counts for the entire analysis were used, as is considered the best practice in bulk RNA sequencing analysis,^32^ at least in cases where RNA sequencing library was prepared without unique molecular identifiers.

Counts were filtered for low-count genes and library sizes were normalized with the trimmed mean of M-values method^33^ via edgeR *filterByExpr* and *normLibSizes* functions. Linear model fit for the comparison of *Mec*r-KO samples with WT was performed with edgeR function *voomLmFit* wrapper function of limma *′voomWithQualityWeights* to combine observational-level precision weights with sample-specific quality weights and increase the power of the analysis.^34^ Volcano plots were visualized with EnhancedVolcano 1.22.0 and interactive volcano plots were customized with ggplot2^35^ 3.5.1 plotly^36^ 4.10.4 and shiny 1.9.1. Gene set enrichment analysis (GSEA)^37^ was performed with clusterProfiler^38–40^ 4.12.6 using fgsea^41^ as the calculation method with no boundaries for *P* value estimation. For the GSEA we included all the genes into the background ranked gene list after excluding the low counts genes with *filterByExpr*, thus leaving the genes that have any chance to be assessed as differentially expressed.^42^^,^^43^ The background gene list was ranked by the moderated *t* statistic. We tested the background gene list against MSigDB′s^44^ via msigdbr 7.5.1 Hallmark pathways.^45^ Pathways with a *q* value <0.05 were deemed to be significant. The integrated network analysis of transcriptional data was performed using Shiny-GATOM.^46^ Raw and processed transcriptomics data are deposed at National Center for Biotechnology Information Gene Expression Omnibus as GSE280350. Data analysis scripts are available at GitHub repository (https://github.com/MogilenkoLabVUMC/MECR_KO_in_vivo-KayLee).

### Quantification and statistical analysis

Graphs and statistical tests were generated using GraphPad Prism 10 (GraphPad Software) unless otherwise noted. Sample sizes were chosen based on previous studies. Statistical significance was performed by unpaired 2-sided *t* tests, Mann-Whitney tests, 2-way analysis of variance with Tukey post hoc test, Dunnett’s post hoc test, Kruskal-Wallis test, or Welch’s *t* test. Graphs show mean and SD unless otherwise stated. A *P* value >0.05 was considered not significant.

### Data availability

Custom guide RNA library information and resulting CRISPR/Cas9 screening data are available on https://functionalimmunogenomics.shinyapps.io/crispr/. Data used for analysis in Fig. S1 were from the Immunological Genome Project database (ImmGen) (Fig. S1A),^47^ GSE232241 (Fig. S1B),^17^ GSE41870 (Fig. S1C),^48^ and Immunological Proteomic Resource (ImmPRes) (Fig. S1D, E).^49^ Raw and processed transcriptomics data generated for this article are deposed at National Center for Biotechnology Information Gene Expression Omnibus as GSE280350.

## Results

### mtFAS genes regulate T cell fitness across multiple in vivo CRISPR/Cas9 screens

**Figure 1.**
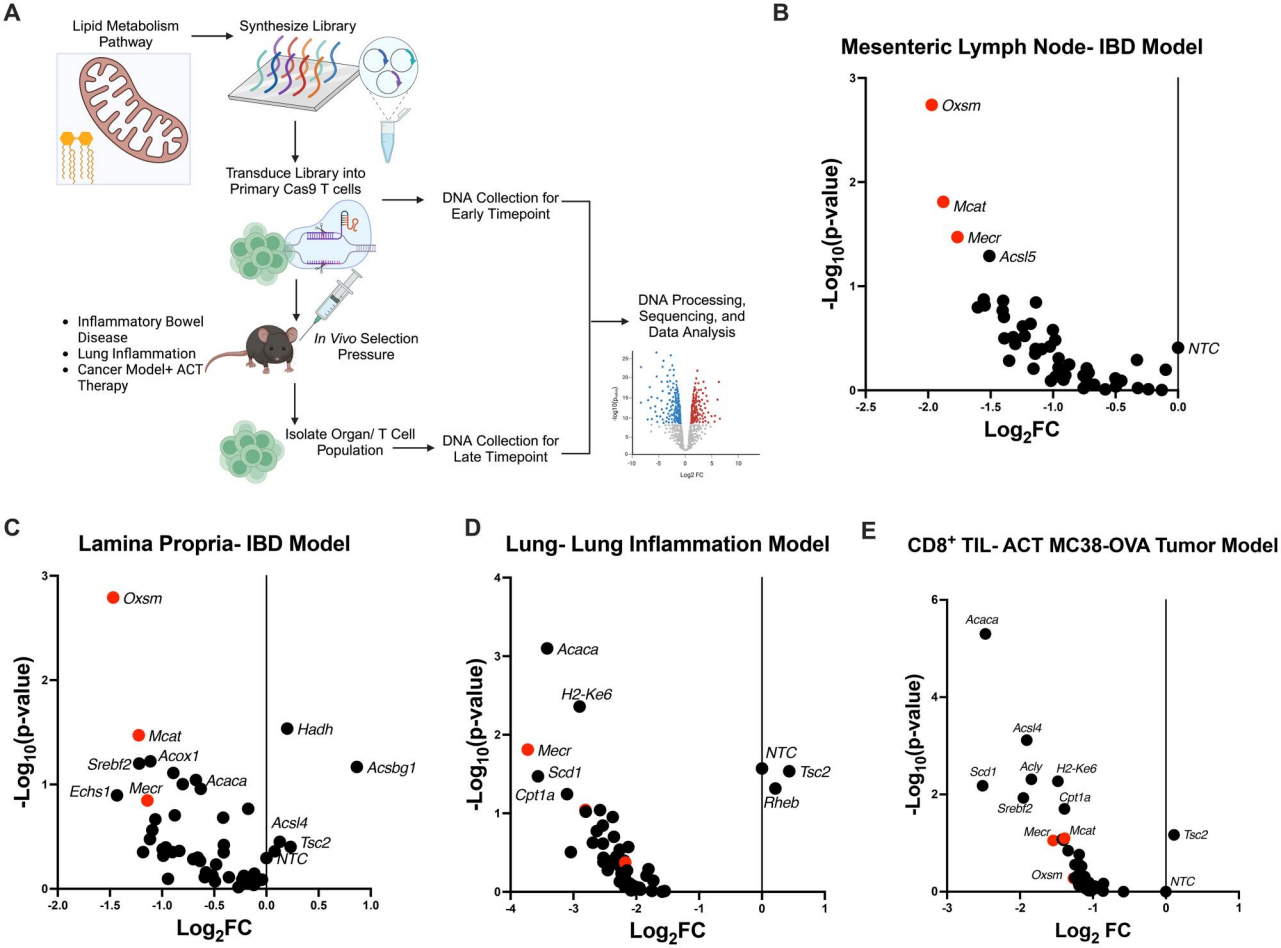
CRISPR/Cas9 in vivo screens utilizing a lipid metabolism targeted guide RNA library identify mtFAS genes. (A) In vivo CRISPR/Cas9 screening protocol. (B–E) Analysis of CRISPR/Cas9 screens in an IBD model (B, C), OT-II lung inflammation (D), and a CD8^+^ adoptive transfer therapy (ACT) MC38-OVA tumor model (E). Statistical significance performed by MAGeCK. Panel A made using Biorender. Panels D and E show the results of a representative experiment of 2 independent experiments. (B) n = 6 biological replicates; (C) n = 4 biological replicates; (D) n = 3 biological replicates; (E) n = 4 biological replicates. FC, fold change.

An in vivo CRISPR screening approach of primary murine T cells was employed in models of inflammation to test the dependence of CD4^+^ and CD8^+^ T cells on lipid metabolism (Fig. 1A). A custom CRISPR/Cas9 library was constructed to target 47 lipid metabolism genes found in the Kyoto Encyclopedia of Genes and Genomes lipid metabolism database, with 10 nontargeting negative controls (NTCs) and guides targeting 2 positive control genes (*Tsc2* and *Rheb*) in a retroviral vector (Table S1). CD4^+^ T cells were activated in Th17-promoting conditions, transduced with the lipid metabolism library, and adoptively transferred into *Rag1^−/−^* mice to induce IBD. Mice were euthanized upon 20% loss of body weight or after 8 wk and CD4^+^ T cells were isolated from the mLNs and the colonic lamina propria by tissue dissociation and magnetic bead sorting. The relative abundance of each guide RNA (sgRNA) from DNA in the T cells of the mLNs and the lamina propria was determined by next-generation sequencing and compared with sgRNA frequencies prior to adoptive transfer. Interestingly, the sgRNAs for genes involved in mtFAS, including *Mecr*, *Oxsm*, and *Mcat*, were consistently depleted in T cells, suggesting a role for mtFAS in T cell fitness in the mLNs and the lamina propria in the IBD model (Fig. 1B, C).

To validate these results, the same lipid metabolism CRISPR screen was performed in additional models. First, an antigen-specific model of lung inflammation was tested. Ovalbumin (OVA)-specific OT-II; Cas9 CD4^+^ T cells were activated and transduced with the lipid-targeting sgRNA library and adoptively transferred into *Rag1^−/−^* mice, which were then intranasally sensitized with OVA protein to promote lung inflammation. T cells recovered from the lung had reduced frequencies of *Mecr*-targeting sgRNAs, showing that the depletion is not disease specific (Fig. 1D). Second, a model of tumor adoptive transfer immunotherapy was utilized to test the dependence in CD8^+^ T cells on mtFAS. Transduced OT-I CD8^+^ T cells were transferred into MC38-OVA tumor-bearing *Rag1^−/−^* mice. After 7 d, CD8 tumor-infiltrating T cells were isolated from tumors for analysis. In this screen, *Mecr* and *Mcat* sgRNAs were depleted in the antigen-specific CD8^+^ T cells in the tumor (Fig. 1E). The depletion of sgRNAs of mtFAS in CRISPR screens across multiple in vivo models supports a role for mtFAS and the genes *Mecr*, *Oxsm*, and *Mcat* in the fitness of both CD4^+^ and CD8^+^ T cells.

Because mtFAS has not been studied in depth in immunity, *Mecr*, *Mcat*, and *Oxsm* expression was examined in immune cells. We first examined murine studies from available RNA sequencing datasets in T cell development and activation. While *Mcat* and *Oxsm* were expressed at low levels across thymic development, *Mecr* was dynamically regulated with high early expression in double negative thymocytes and drastic reduction in double positive thymocytes. In addition, subsequent naïve and activated T cells have increased *Mecr* expression (Fig. S1A).^50^ In CD8^+^ T cells, *Mecr* expression was increased after activation in mature murine antigen specific OT-I CD8^+^ T cells (Fig. S1B).^17^ In CD8^+^ T cells in LCMV Armstrong acute infection, *Mecr* expression initially increased after 6 d but then decreased to normal as the response was complete (Fig. S1C). However, during a chronic LCMV clone 13 infection, chronically stimulated and exhausted T cells decreased and maintained a low *Mecr* expression over the course of 30 d.^48^ MECR protein expression was highly expressed in both human CD8^+^ and CD4^+^ T cells, compared with MCAT and OXSM protein expression (Fig. S1D).^49^ Based on these expression profiles, MECR was selected for further study.

### *Mecr* ^fl/fl^; *Cd4*^cre^ mice have reduced CD4^+^ and CD8^+^ T cells ex vivo

**Figure 2.**
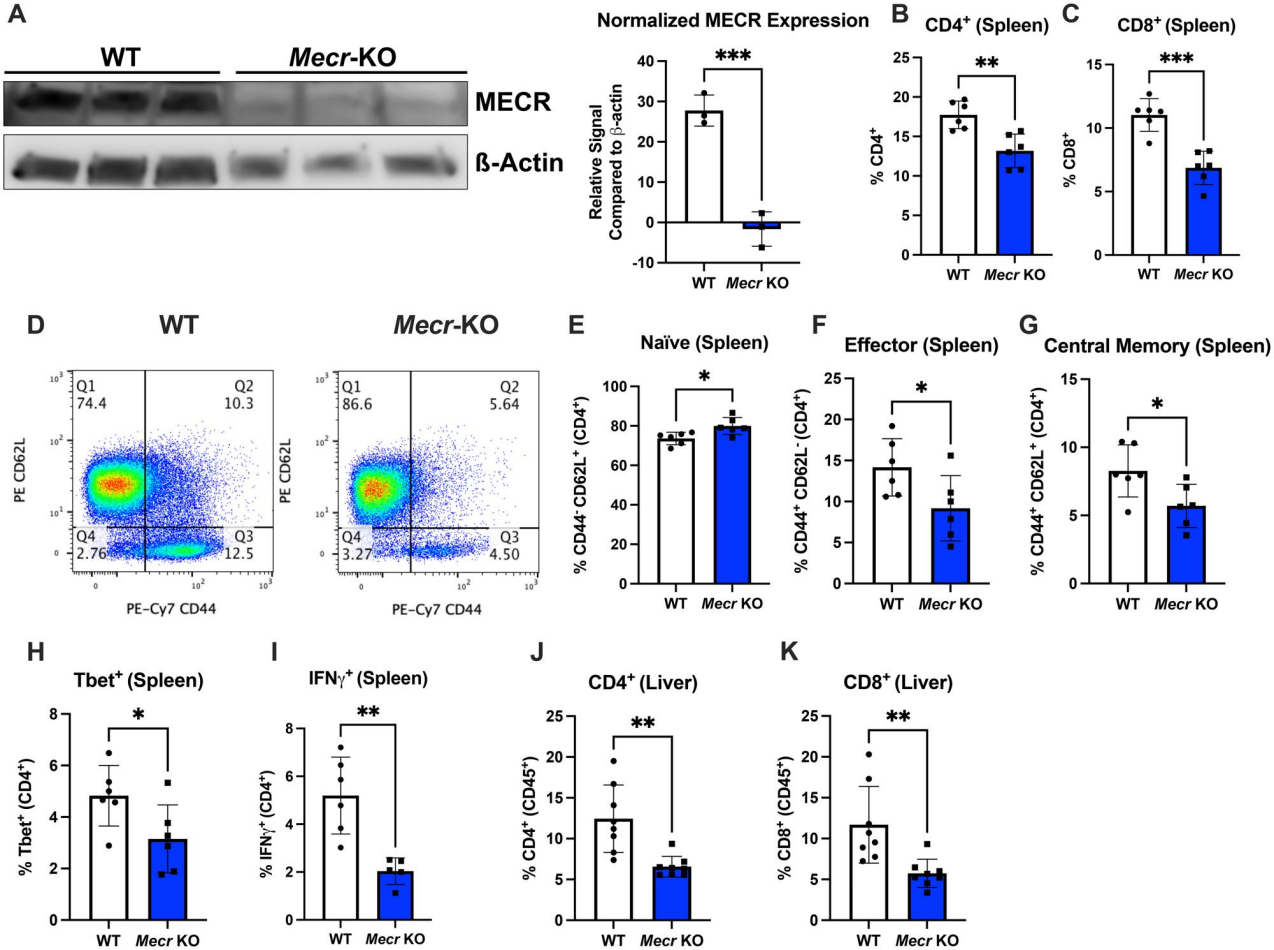
*Mecr* ^fl/fl^; *Cd4*^cre^ mice have reduced CD4 and CD8 T cells ex vivo. (A) Western blot of MECR (40 kDa) expression in activated CD4^+^ T cells and quantification compared with β-actin (42 kDa). (B) Percentage of inactivated CD4^+^ T cells in the spleen measured by flow cytometry. (C) Percentage of inactivated CD8^+^ T cells in the spleen. (D–G) Naïve, effector, and central memory CD4^+^ T cells in the spleen. (H, I) Th1 function percentage of Tbet and IFNγ^+^. (J, K) Percentage of CD4^+^ and CD8^+^ in the liver. Panels B–K show results from 2 pooled independent experiments. Each data point represents a biological replicate and error bars show standard deviation. All Statistical significance performed by unpaired *t* tests. *P < 0.05, **P < 0.01, ***P < 0.001.

Because chronic loss of MECR may be required to reveal the full role of mtFAS in T cells, we developed a CD4^+^ and CD8^+^ T cell specific conditional *Mecr*-KO mouse model (*Mecr^fl/fl^*; *Cd4^cre^* mice). CD4^+^ T cells were activated and MECR appeared reduced in *Mecr*-KO T cells by Western blot (Fig. 2A), although some changes in activation may have affected beta-actin levels. All following experimental flow cytometry gating was analyzed on live singlet CD4^+^ T cells (Fig. S2A). When analyzing resting T cells, the percentages of thymocyte double negative, double positive, and CD4 and CD8 single positive cell populations were unchanged (Fig. S2B–E). Total numbers of live splenocytes between control Cre− (WT) and Cre+ (*Mecr*-KO) T cells (Fig. S2F) and the total number of peripheral CD4^+^ and CD8^+^ T cells were also unchanged (Fig. S2G, H). However, the percentages of CD4^+^ and CD8^+^ T cells in the spleen were significantly reduced (Fig. 2B, C). Interestingly, the CD4^+^ T cell compartment of MECR-deficient mice skewed toward an increased proportion of naïve cells and reduced fraction of effector (CD62L^−^ CD44^+^) and central memory (CD62L^+^ CD44^+^) CD4^+^ T cells (Fig. 2D–G). MECR-deficient T cells also had decreased basal percentages of Tbet^+^ cells and IFNγ^+^ CD4^+^ T cells (Fig. 2H, I), suggesting a defect in Th1 effector T cells. In contrast, Treg populations appeared normal (Fig. S2I, J). Livers were also analyzed as a representative tissue and CD4^+^ and CD8^+^ T cell percentages were also decreased (Fig. 2J, K), showing that *Mecr*-KO mice have a systemic decrease in these T cell subsets. MECR, therefore, was not critical for naïve T cells or their homing to the spleen but was required for full activation of effector memory populations and tissue homing or persistence.

### MECR is required for efficient CD4^+^ T cell proliferation and differentiation

**Figure 3.**
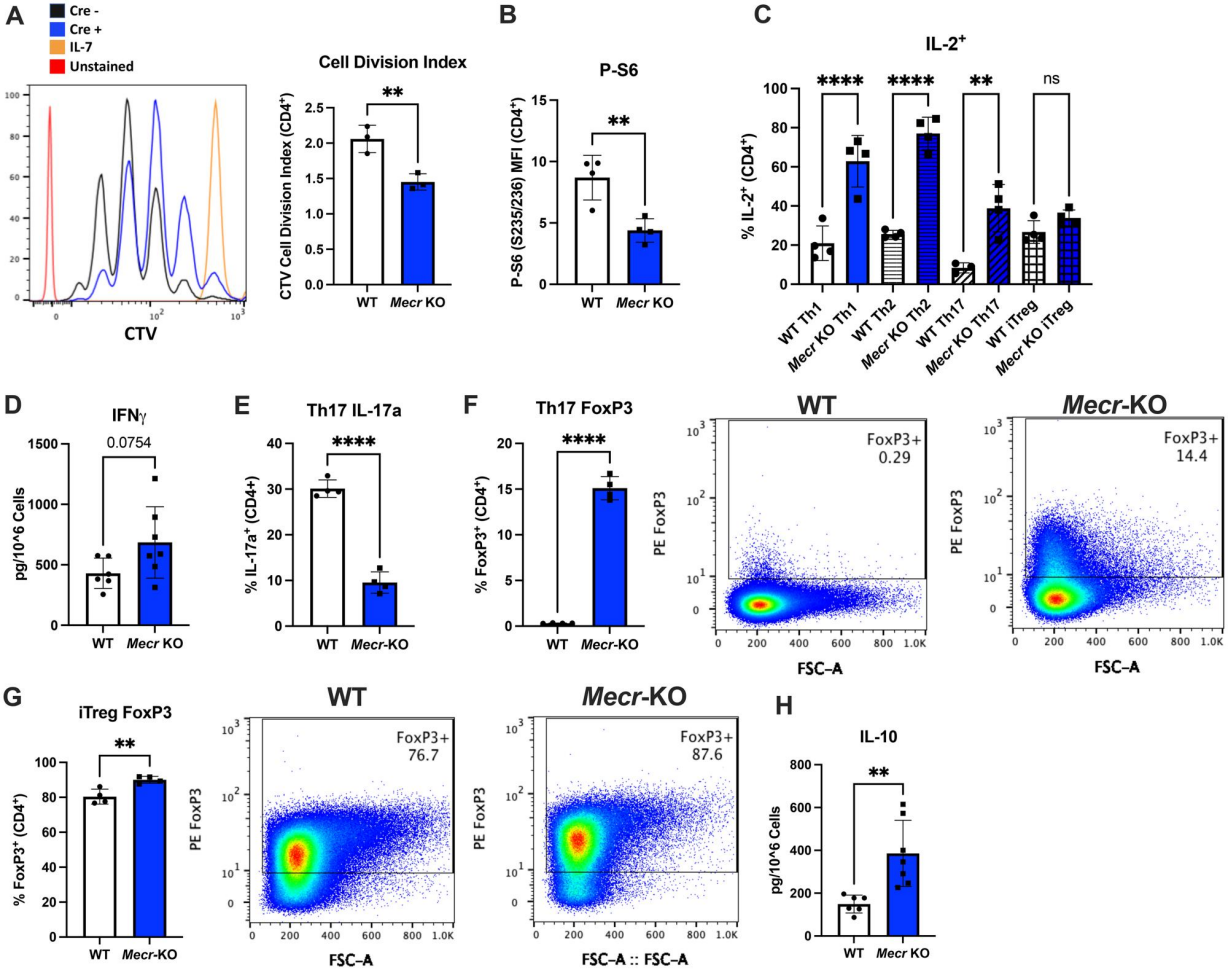
MECR is required for T cell proliferation and differentiation. (A) Cell division index of activated CD4^+^ T cells stained with CTV measured by flow cytometry. (B) Phospho-S6 mean fluorescence intensity (MFI) of activated CD4^+^ T cells. (C) Percentage of IL-2^+^ T cells in each T cell subset. (D–H) Differentiated naïve CD4^+^ T cells. (D) cytokine production of Th1 IFNγ. (E) Percentage of Th17 IL-17a^+^. (F) Percentage of Th17 FoxP3^+^. (G) Percentage pf iTreg FoxP3^+^. (H) Cytokine production of iTreg IL-10. Panels A and B show representative results from 3 independent experiments. Panels C, E, F, and G show representative results from 6 independent experiments, and in panels D and H data are representative of pooled results of 2 independent experiments. All panels were measured by flow cytometry. Each data point represents a biological replicate and error bars show standard deviation. All statistical significance was performed by unpaired *t* tests. **P < 0.01, ****P < 0.0001. FSC-A, forward scatter area; ns, not significant.

We next tested the effects of chronic loss of MECR on CD4^+^ T cell proliferation, survival, and differentiation. CD4^+^ T cells were isolated from *Mecr^fl/fl^*; *Cd4^cre^* mice and activated with anti-CD3, anti-CD28, and IL-2 while stained with CTV. After 3 d, *Mecr*-KO T cells had significantly reduced proliferation as measured by the cell division index of CTV dilution compared with the cells from WT mice (Fig. 3A). The mTORC1 pathway responds to available nutrients and has multiple downstream metabolic mechanisms to promote cell growth and proliferation by increasing nucleotide, protein, and lipid synthesis. To test if the effects of MECR on proliferation are linked to mTORC1 activity, we stained activated *Mecr*^fl/fl^; *Cd4*^cre^ CD4^+^ T cells for phospho-S6, S235/236, as a measurement of mTORC1 activity. *Mecr*-KO cells had significantly reduced P-S6 mean fluorescence intensity compared with controls (Fig. 3B), although potential changes in total levels of mTORC1 components may influence this finding. Therefore, MECR appears essential to promote or maintain mTORC1 activity.

To test if MECR impacts T cell differentiation in vitro, naïve CD4^+^ T cells were isolated from *Mecr^fl/fl^;*  *Cd4^cre^* mice and activated with cytokines for Th1, Th2, Th17, or iTreg polarization. After 3 d, the ability for cells to differentiate was measured by lineage specific transcription factors and cytokines along with CTV. Proliferation was reduced in all MECR-deficient subsets, with Th17 and iTregs the most impacted, followed by Th1 then Th2 (Fig. S2K). MECR-deficient cells polarized in proinflammatory Th1, Th2, or Th17 conditions were more likely to produce IL-2, suggesting that the loss of MECR may impact the differentiation of 1 or more of these cell subsets (Fig. 3C). To confirm this, we quantified lineage specific transcription factors and cytokines for CD4 T cell subsets. While IFNγ production trended to increase in Th1 cells, there was no significant difference in Tbet^+^ T cells (Fig. 3D; Fig. S2L). There was also no significant changes in the Th2-associated transcription factor Gata3 (Fig. S2M), indicating that acute loss of MECR does not affect the differentiation of CD4^+^ T cells into the Th1 or Th2 subset. Interestingly, Th17 cells showed a significant decrease in IL-17a^+^ cells, and a significant increase in FoxP3^+^ T cells (Fig. 3E, F), suggesting that Th17 cells may adopt a suppressive phenotype in vitro. In addition, the iTreg associated transcription factor FoxP3 showed an elevated trend with MECR deficiency (Fig. 3F), and IL-10 production was increased in MECR-deficient iTregs as measured by enzyme-linked immunosorbent assay (Fig. 3G). These data show that MECR-mediated metabolism broadly supports CD4 T cell proliferation.

### MECR deficiency reduces mitochondrial function and oxidative metabolism

**Figure 4.**
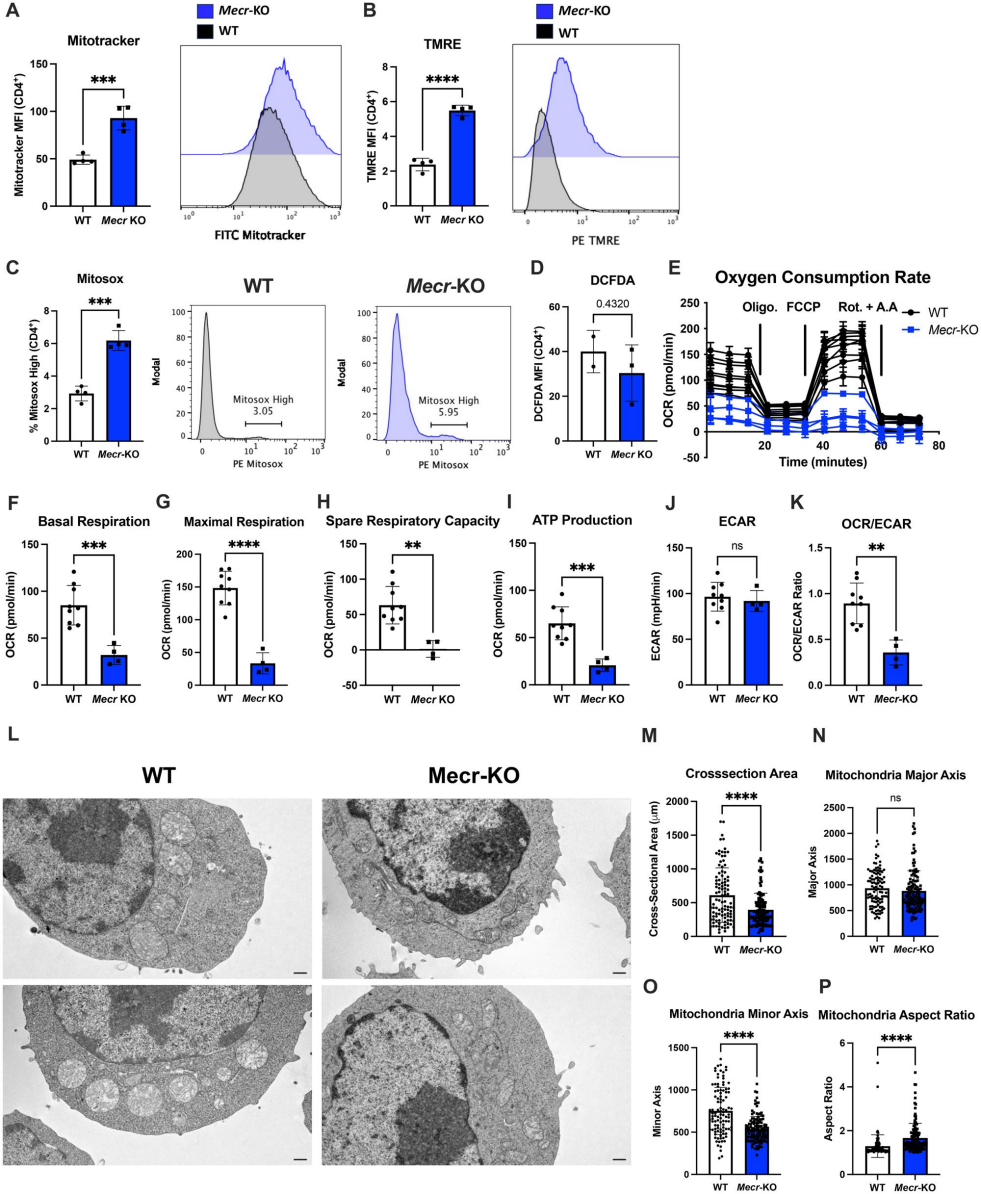
*Mecr*-KO causes reduced mitochondrial function. (A–D) Mitochondrial stains in activated CD4^+^ T cells from WT and *Mecr*-KO mice by flow cytometry, MitoTracker (A), TMRE (B), MitoSOX (C), and DCFDA (D). (E) Extracellular flux analysis on activated CD4^+^ T cells. (F, G) Basal and maximal respiration. (H) Spare respiratory capacity. (I) ATP production. (J) Extracellular acidification rate (ECAR). (K) Oxygen consumption rate (OCR)/extracellular acidification rate (ECAR) ratio. (L) Transmission electron microscopy (TEM) of activated CD4^+^ T cells. Scale bars represent 500 nm. (M) Cross-sectional area. (N) Mitochondrial major axis. (O) Mitochondrial minor axis. (P) Mitochondrial aspect ratio. Panels A–D show representative results from 2 independent experiments and each data point represents a biological replicate. Panels E–K show results from 2 pooled independent experiments. For extracellular flux analysis, each data point represents a biological replicate with averaged n > 3 technical replicates. Panels L–P show representative results from 2 independent experiments. Error bars show standard deviation. All statistical significance was performed by unpaired *t* tests. **P < 0.01, ***P < 0.001, ****P < 0.0001. MFI, mean fluorescence intensity.

*Mecr*-KO skeletal myoblast cells have been reported to have disrupted mitochondrial function.^13^ To test if this also occurred in T cells, activated *Mecr*^fl/fl^; *Cd4*^cre^ CD4^+^ T cells were stained for mitochondrial function, including mitochondrial mass (MitoTracker), mitochondrial membrane potential (TMRE), and mitochondrial ROS (mtROS) (MitoSOX) after 3 d postactivation. MitoTracker and TMRE were significantly increased in MECR-deficient cells (Fig. 4A, B) suggesting increased mitochondrial size and dysregulation of the ETC proton gradient. In addition, mtROS was significantly increased in activated *Mecr*-KO CD4^+^ T cells (Fig. 4C). However, total ROS measured by DCFDA was unchanged. Altered ROS was thus a mitochondrial specific effect or was masked by cytosolic adaptations (Fig. 4D).

Dysregulated mitochondrial function with MECR deficiency caused by reduced ETC assembly or stability may reduce oxidative metabolism, as acyl-ACP–dependent Leu-Tyr-Arg motif–containing proteins (LYRM) that maintain ETC complexes can decrease in the absence of mtFAS.^13^ To test if respiration defects were observed in *Mecr*-KO CD4^+^ T cells, we conducted a mitochondrial stress test to analyze T cell mitochondrial function (Fig. 4E). Indeed, the basal and maximal respiration of the MECR-deficient CD4^+^ T cells were significantly reduced, spare respiratory capacity was nearly eliminated, and adenosine triphosphate (ATP) production was reduced when compared with WT counterparts (Fig. 4F–I). In contrast, measurement of the basal extracellular acidification rate, which reflects the generation of lactate, was unchanged in MECR-deficient T cells (Fig. 4J) while the selective decrease in oxygen consumption rate while extracellular acidification rate was maintained (Fig. 4K). With dysregulated mitochondria function and respiration, these data show that MECR is needed for efficient ATP-linked mitochondrial respiration and shows a relative shift toward glycolytic metabolism.

To visualize mitochondrial defects, transmission electron microscopy was performed on activated WT or *Mecr*-KO CD4^+^ T cells. Mitochondria from *Mecr*-KO T cells were found to be more fragmented or elongated (Fig. 4L). Additionally, the cross-sectional area of mitochondria from *Mecr*-KO T cells was significantly reduced compared with WT controls (Fig. 4M). Last, mitochondria from *Mecr*-KO T cells were more tubular shaped, with no significant differences in the mitochondria major axis but a significant decrease in the minor axis. This was reflected in a significant increase in the aspect ratio of *Mecr*-KO mitochondria (Fig. 4N–P). These results show that MECR deficiency in activated T cells leads to broadly dysfunctional mitochondria morphology and oxidative metabolism.

### CD4^+^ T cells lacking MECR have impaired TCA cycling and ETC metabolism

**Figure 5.**
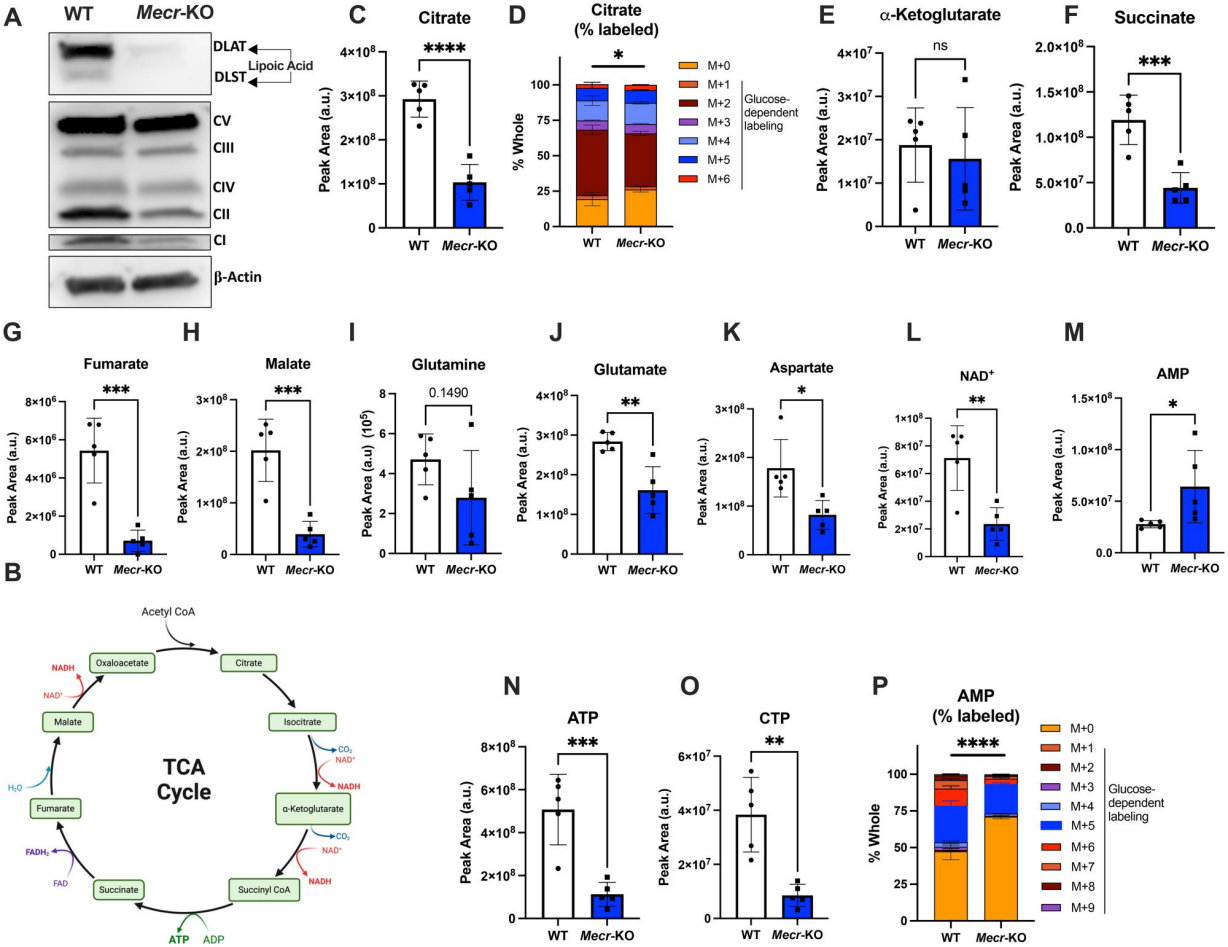
*Mecr*-KO T cells have reduced oxidative metabolism. (A) Western blot of activated CD4^+^ T cells blotted for lipoic acid on PDH complex DLAT (69 kDa) and OGDH complex DLST (50 kDa), ETC complexes I–V (20, 30, 48, 40, and 55 kDa, respectively), and β-actin (42 kDa). (B) TCA cycle. (C–O) WT and *Mecr*-KO CD4^+^ T cells were activated for 3 d before incubation with ^13^C_6_ glucose for 4 h. Cells were lysed and analyzed by metabolomics. (C) Relative peak area of citrate. (D) Percent of citrate. (E–K) Relative peak area of α-ketoglutarate (E), succinate (F), fumarate (G), malate (H), glutamate (I), aspartate (J), NAD^+^ (K), and adenosine monophosphate (AMP) (L). (M) Percent of AMP. (N) Relative peak area of ATP. (O) Relative peak area of cytidine triphosphate. Panel B made using Biorender. Panels A–O show results from 1 independent experiment. Each data point represents a biological replicate and error bars show standard deviation. (C–O) Statistical significance performed by Welch’s *t* test. *P < 0.05, **P < 0.01, ***P < 0.001, ****P < 0.0001. ADP, adenosine diphosphate; ns, not significant.

To further evaluate mitochondrial function, ETC complex proteins were analyzed by immunoblot in activated CD4^+^ T cells. ETC complex assembly and stability may rely on MECR through acyl-ACP and complexes I and II were most reduced in *Mecr*-KO T cells (Fig. 5A), confirming ETC defects. Acyl-ACP can also be used to synthesize lipoic acid, which is essential for the activity of PDH and OGDH. Using a commercial antibody to probe for lipoic acid, we found an almost complete loss of lipoic acid on PDH complex DLAT and OGDH complex DLST in MECR-deficient T cells (Fig. 5A). MECR, therefore, appears needed for ETC complexes and lipoic acid synthesis for lipoylated enzymes.

We next directly analyzed the abundance of TCA intermediates and carbon source utilization in activated CD4^+^ T cells from *Mecr*^fl/fl^; *Cd4*^cre^ mice. T cells were cultured with uniformly labeled ^13^C glucose for 4 h followed by mass spectrometry to measure metabolite abundance and to trace ^13^C flux (Fig. 5B). The total amount of citrate was significantly reduced in the *Mecr*-KO, with the percent of ^13^C-labeled citrate significantly decreased in the KO compared with the control (Fig. 5C, D). Interestingly, while the total amount of α-ketoglutarate was unchanged (Fig. 5E). Succinate, fumarate, and malate that follow in the TCA cycle were all decreased (Fig. 5F–H). Similarly, glutamine was unchanged (Fig. 5I), but glutamate and aspartate were each decreased (Fig. 5J, K). Importantly, NAD^+^ was decreased (Fig. 5L) suggesting decreased capacity to convert NAD^+^ to NADH for use by the ETC to produce ATP. Consistent with impaired TCA flux and oxidative phosphorylation, *Mecr*-KO T cells had increased total adenosine monophosphate (AMP), but decreased ATP and cytidine triphosphate (Fig. 5M–O). This increase in AMP was not due to new synthesis because AMP labeling by 13C was reduced in *Mecr*-KO T cells (Fig. 5P). Thus, MECR is essential to maintain oxidative metabolism and TCA cycle intermediates in activated CD4^+^ T cells.

### *Mecr*-KO T cells accumulate iron and are susceptible to ferroptosis

**Figure 6.**
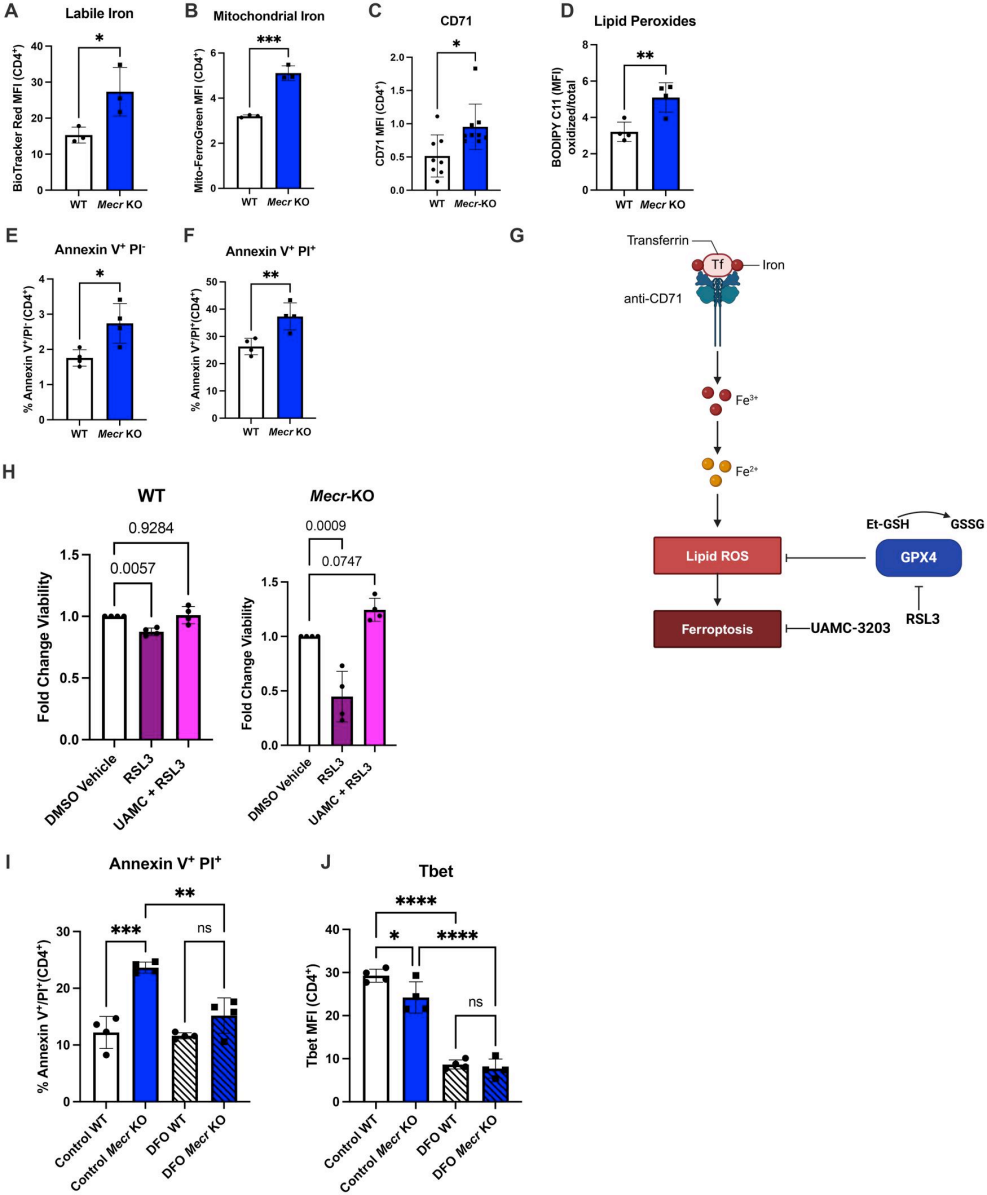
*Mecr*-KO T cells accumulate iron, which causes increased sensitivity to ferroptosis. (A–C) Activated CD4^+^ T cells stained for labile (2+) iron (A), mitochondrial iron (B), transferrin receptor (CD71) (C), and C11 BODIPY (D). (E, F) Percentage of apoptosis (E) and cell death (F) of activated T cells. (G) Pathway of ferroptosis. (H) Viability change of activated T cells with treatment of RSL3 compared with dimethyl sulfoxide (DMSO) vehicle control. Each cell type was calculated based on the starting viability of the DMSO control. (I, J) Percentage of cell death (I) and T-bet mean fluorescence intensity (MFI) (J) when treated with iron chelator DFO. All experiments were measured by flow cytometry. Panels A–D show representative results from 3 independent experiments. Panels H–J show results from 1 independent experiment. Panels E and F show results from 2 independent experiments. Panel G made using Biorender. Each data point represents a biological replicate and error bars show standard deviation. (A–F) Statistical significance performed by unpaired *t* tests. (H) Statistical significance performed by 1-way analysis of variance with Dunnett’s multiple comparison. (I, J) Statistical significance performed by 2-way analysis of variance with Tukey’s multiple comparison. *P < 0.05, **P < 0.01, ***P < 0.001, ****P < 0.0001. ns, not significant.

MECR-deficient cells from *Drosophila* and human MEPAN patient fibroblasts accumulate excess iron and ceramides due to defective mitochondrial Fe-S cluster biogenesis.^15^ T cell subsets have different requirements for iron, with Th1 being more dependent on iron than Th2,^51^ and Th17 cells requiring iron for differentiation and IL-17a production.^52^ Further, blocking iron uptake by neutralizing the transferrin receptor (CD71) alters mitochondrial function and OXPHOS in murine and human T cells.^53^ Therefore, we hypothesized that *Mecr*-KO T cells may have dysregulated iron metabolism that impaired mitochondrial respiration. CD4^+^ T cells from *Mecr*^fl/fl^; *Cd4*^cre^ mice were activated for 3 d and cellular labile ferrous iron (II) mitochondrial iron and transferrin receptor (CD71) were measured. All were significantly increased in MECR-deficient T cells compared with control cells (Fig. 6A–C). In addition, lipid peroxidation was increased in T cells lacking MECR based on C11 BODIPY staining (Fig. 6D), and the percent of cell death in activated T cells was significantly increased (Fig. 6E, F). These demonstrate that loss of MECR increased levels of iron, lipid peroxidation, and cell death.

Because T cells showed both increased iron and cell death, we hypothesized that the observed increase in cell death may be caused by ferroptosis (Fig. 6G). To test this, activated WT and *Mecr*-KO T cells were cultured for 3 d and cell treatments were challenged with the GPX4 inhibitor RSL3 to specifically promote ferroptotic cell death. Some groups were also pretreated with ferroptosis inhibitor UAMC-3203 to rescue cell death caused by RSL3.^54^  *Mecr*-KO cells had reduced viability when treated with ferroptosis inducer RSL3 compared with WT cells, demonstrating increased susceptibility to ferroptosis (Fig. 6H). Importantly, *Mecr*-KO cell viability was rescued when treated with the specific ferroptosis inhibitor UAMC-3203. These data show that *Mecr*-KO cells accumulate iron and lipid peroxidation, which may contribute to increased sensitivity to cell death by ferroptosis. In addition, cells were treated with iron chelator DFO to test if this could also rescue the phenotypes of *Mecr*-KO cells. While WT cells appeared unaffected by this dose of DFO, *Mecr*-KO cells were partially rescued from cell death upon treatment with DFO (Fig. 6I). However, both WT and KO cells treated with DFO showed reduced T-bet (Fig. 6J), showing reduced function of the treated cells. These results show that *Mecr*-KO T cells accumulate iron which are susceptible to ferroptosis and can be partially rescued by ferroptotic inhibitors and iron chelators.

### Loss of MECR reduces T cell survival and function in IBD model of colitis in vivo

**Figure 7.**
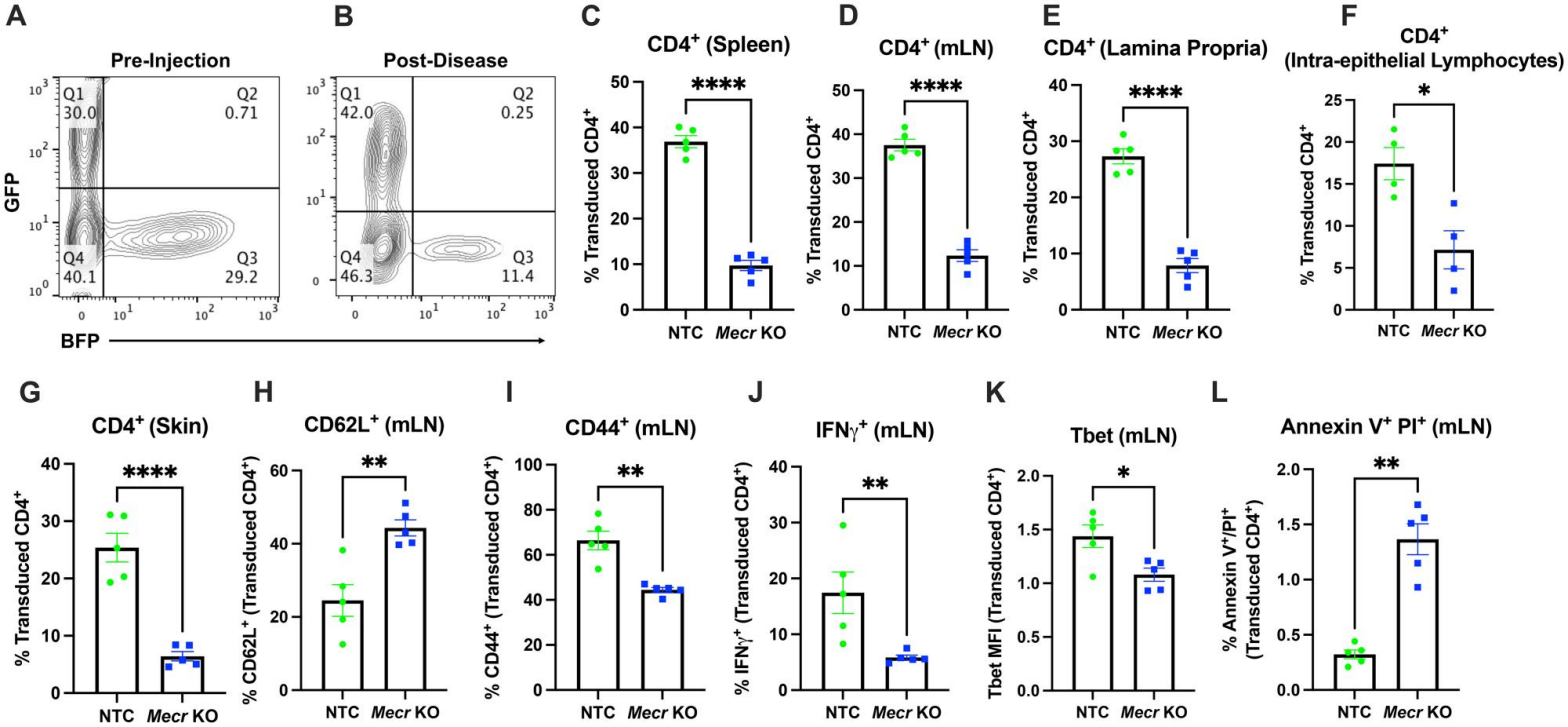
*Mecr*-KO causes reduced T cell survival in an in vivo model of IBD. (A) The 1:1 mixed sgNTC T cells (GFP) and sg*Mecr* (BFP) preinjection. (B) GFP^+^ and BFP^+^ T cells of CD4^+^ T cells post–IBD development. (C–G) Percentage of transduced CD4^+^ expressing GFP (NTC) or BFP (*Mecr*-KO) in the spleen by flow cytometry (C), mLN (D), lamina propria (E), intraepithelial lymphocytes (F), and skin (G). (H) CD62L^+^ of transduced CD4^+^ T cells. (I) CD44^+^ of transduced CD4^+^ T cells. (J) IFNγ^+^ of transduced CD4^+^ T cells. (K) T-bet mean fluorescence intensity of transduced CD4^+^ T cells. (L) Annexin V^+^ Propidium Iodide (PI)^+^ of transduced CD4^+^ T cells. All panels were measured by flow cytometry. (A–L) Representative results from >5 independent experiments. Each data point represents a biological replicate and error bars show standard error of the mean. All statistical significance was performed by unpaired *t* tests with the exception of panels I, J, and L with a Mann-Whitney test. *P < 0.05, **P < 0.01, ****P < 0.0001.

Because *Mecr* was consistently depleted across several in vivo CRISPR screens and promoted mitochondrial metabolism of activated T cells, we hypothesized that *Mecr*-KO T cells would have reduced fitness and effector differentiation during disease development. Th17-skewed Cas9-transgenic CD4^+^ T cells were transduced with NTC sgRNA in a GFP-expressing retroviral vector or a *Mecr*-targeting sgRNA in a BFP-expressing retroviral vector shown to induce knockout (Fig. S3A). Cells were mixed at a 1:1 ratio (Fig. 7A; Fig. S3A) and injected into *Rag1*^−/−^ mice to induce IBD. Animals were analyzed after 8 wk or at 20% body weight loss. Prior to injection, there was no difference in expression of the Th17 transcription factor, RORγt expression (Fig. S3B). After 8 wk, the transduction efficiency was analyzed by flow cytometry. The frequency of *Mecr*-KO CD4^+^ T cells (BFP) was significantly reduced in the spleen, mLNs, lamina propria, intraepithelial lymphocytes, and the skin compared with control NTC cells (GFP) (Fig. 7B–G), confirming that MECR loss reduces survival or recruitment of activated T cells. *Mecr*-KO cells that were present had increased surface expression of CD62L and decreased CD44 (Fig. 7H, I). These data suggest that *Mecr*-KO T cells have a reduced capacity to survive and that the remaining T cells may have reduced activation or effector function.

To further investigate *Mecr*-KO T cell function and phenotypes, cytokine and transcription factors were quantified. Th17-skewed cells can show plasticity to a Th1 phenotype in colitis, as previously published.^55^ With this IBD model, *Mecr*-KO T cells had a significantly reduced percentage of IFNγ^+^ T cells and reduced Tbet expression that was consistent with impaired Th1 function when compared with the NTC T cells (Fig. 7J, K). In addition, the percentages of IL-17a^+^ IFNγ^+^ and IL-17a^+^ IFNγ^−^ T cells showed a trend to decrease (Fig. S3C–E). *Mecr*-KO T cells also exhibited increased cell death (Annexin V^+^ PI^+^) compared with NTC transduced T cells (Fig. 7L). These data show that the loss of MECR reduced CD4^+^ T cell survival and Th1 function in vivo.

### MECR is required for CD4^+^ T cell-driven colitis development

**Figure 8.**
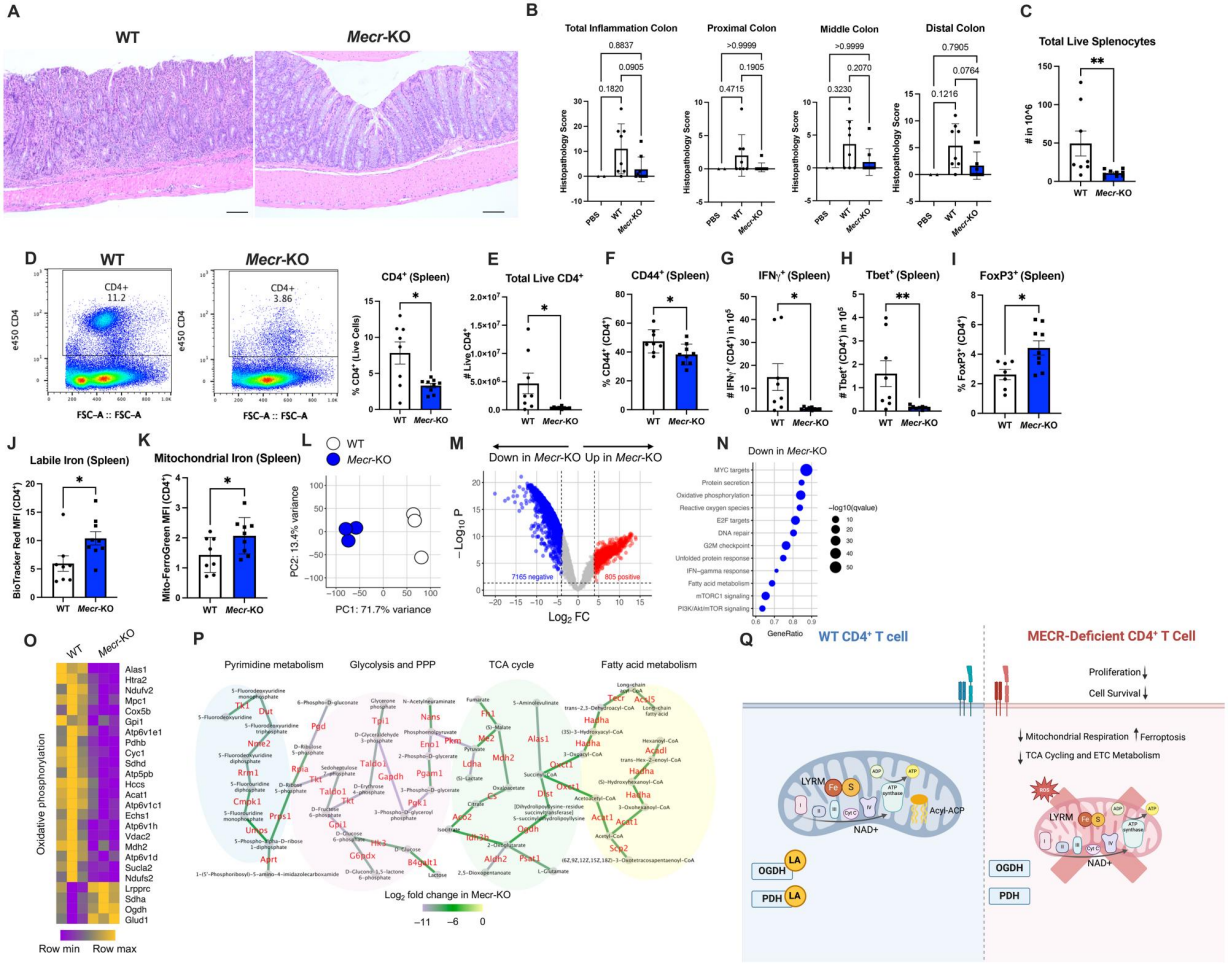
*Mecr*-KO causes reduced inflammation and disease severity in an in vivo model of IBD. (A) Hematoxylin and eosin of colons from *Rag1^−/−^* mice with WT or Mecr-KO CD4^+^ T cell transfer. (B) Histopathology score of total colon, proximal colon, middle colon, and distal colon from a board-certified pathologist. (C) Total number of live splenocytes in 10^6^. (D) Percentage of CD4^+^ T cells from the spleen. (E) Number of total live CD4^+^ T cells. (F) Percentage of CD44^+^ CD4^+^ T cells. (G) Total number of IFNγ^+^ CD4^+^ T cells. (H) Total number of Tbet^+^ CD4^+^ T cells. (I) Percentage of FoxP3+ T cells. (J) Mean fluorescence intensity (MFI) of labile iron. (K) MFI of mitochondrial iron. (L) Principal component (PC) analysis of bulk RNA sequencing of adoptively transferred splenic CD4^+^ T cells isolated from *Rag1^–/–^* mice in a model of IBD; n = 3 mice per genotype. (M) Volcano plot showing differential expression of genes between WT and *Mecr*-KO CD4^+^ T cells in vivo; numbers of genes that were up- or down-regulated with the false discovery rate *q* value < 0.05 and log_2_ fold change cutoff > 4 or < –4 are shown. (N) Top “Hallmark” pathways significantly downregulated in *Mecr*-KO compared with WT CD4^+^ T cells; fractions of genes from each pathway enriched (GeneRatio) and the false discovery rate *q* values are shown. (O) Expression levels of the most differentially expressed genes from the OXPHOS pathway in WT and *Mecr*-KO CD4^+^ T cells in vivo. (P) Integrated network analysis of transcriptional and metabolic data using Shiny GATOM^46^ showing connected metabolic pathways containing 50 most differentially expressed metabolic genes whose expression levels were significantly decreased in *Mecr*-KO compared with WT CD4^+^ T cells in vivo (adjusted *P* values <6.6 × 10^−13^). Edges of the graph represent differentially regulated genes and are color coded according to the fold change in their expression levels. Nodes of the graph represent metabolites that are produced by the enzymes encoded by these genes. (Q) Overall conclusion figure. Panels A–P show results from 1 independent experiment. Panels D–K were conducted by flow cytometry. Panel Q was made using BioRender. Each data point represents a biological replicate and error bars show SEM. Statistical significance of panels C–E, G, H, and J are with a Mann-Whitney test, panels F and I with an unpaired *t* test, and panel B with a 1-way analysis of variance with Dunnett’s multiple comparison or Kruskal-Wallis test. *P < 0.05, **P < 0.01. FC, fold change; FSC-A, forward scatter area.

We next tested if *Mecr*-KO T cells were able to independently drive colitis development in a T cell transfer IBD model. Either isolated naïve *Mecr*-KO or WT CD4^+^ T cells were transferred into *Rag1^−/−^* recipients independently. Mice were euthanized when mice lost 20% of their body weight or at 8 wk postinjection, and the colons were prepared for histology and spleens for flow cytometry analysis. After disease development, notably, the colons of mice with *Mecr*-KO T cell transfers had less severe inflammation scores in the total colon and in the proximal, middle, and distal colon (Fig. 8A, B). In addition, the number of splenocytes and CD4^+^ T cells were significantly lower in *Mecr*-KO T cell transfer mice, consistent with reduced inflammation (Fig. 8C–E). Of those few cells remaining, *Mecr*-KO T cells had reduced percentages of CD44^+^ T cells (Fig. 8F) and reduced Th1 phenotypes with fewer IFNγ^+^, Tbet^+^, and IL-2^+^ T cells (Fig. 8G, H; Fig. S3G), and an increase in FoxP3+ (Fig. 8I) showing a reduction in activated proinflammatory T cells in vivo. Last, *Mecr*-KO cells also displayed increased labile iron and mitochondrial iron, suggesting potential increased sensitivity to ferroptosis in vivo, validating our in vitro work, and pointing to a possible mechanism (Fig. 8I, J). These data show that MECR-deficient CD4^+^ T cells are less fit and have reduced capacity to drive immunopathology in an IBD model.

### Transcriptomic analysis shows dysregulated metabolism and cell cycle pathways in MECR-deficient CD4^+^ T cells

To further investigate what pathways are dysregulated in the surviving *Mecr*-KO T cells in the IBD model, CD4^+^ T cells were isolated from the spleens of *Rag1^−/−^* mice after disease development. Bulk RNA was isolated from WT or *Mecr*-KO CD4^+^ T cells and sequenced for transcriptomics analysis. Principal component analysis revealed profound differences between the WT and *Mecr*-KO T cells, showing a clear separation between the genotypes (Fig. 8K). Differential gene expression analysis showed 805 genes that were significantly upregulated and 7,165 genes that were downregulated in the *Mecr*-KO when a cutoff of 16 fold-change increase or decrease was selected (Fig. 8L). Of the most significantly downregulated Hallmark pathways, metabolic pathways such as OXPHOS, fatty acid metabolism, mTORC1 signaling, and PI3K/AKT/mTOR signaling were reduced in the *Mecr*-KO cells (Fig. 8M). Pathways involved in regulating the cell cycle such and genome integrity, such as MYC targets, E2F targets, DNA repair, and G2M checkpoint, were reduced in *Mecr*-KO CD4^+^ T cells. Interestingly, the Th1-associated IFNγ response pathway was also reduced, in accordance with decreased expression of IFNγ and T-bet proteins in *Mecr*-KO CD4^+^ T cells in the in vivo models.

Because the OXPHOS pathway was globally downregulated in *Mecr*-KO CD4^+^ T cells, we confirmed that multiple genes encoding the TCA cycle enzymes and components of the ETC were among the most differentially expressed genes. Expression of these genes were significantly lower in *Mecr*-KO compared with WT CD4^+^ T cells (Fig. 8N), confirming that MECR supports the expression of the OXPHOS pathway components. To gain more integrated insight into the global transcriptional effect of MECR on metabolic networks, we preformed the integrated metabolic network analysis of transcriptional and metabolic data using Shiny GATOM.^46^ We identified connected metabolic pathways represented by the 50 most differentially expressed metabolic genes whose expression levels were significantly and profoundly decreased in *Mecr*-KO compared with WT CD4^+^ T cells in vivo (Fig. 8O). These results identified pyrimidine metabolism, glycolysis and the pentose phosphate pathway, the TCA cycle, and fatty acid metabolism as central metabolic modules whose expression was decreased in the absence of MECR activity in CD4^+^ T cells in vivo (Fig. 8O). These data confirm that *Mecr*-KO CD4^+^ T cells have dysregulated oxidative metabolism and that *Mecr*-KO cells surviving in vivo have dysregulated metabolic processes, IFNγ responses, and cell cycle pathways that can influence their response to activation, inflammation, and disease progression (Fig. 8P).

## Discussion

T cells reprogram lipid synthesis upon activation to support growth and signaling.^1^^,^^3^^,^^5^ In this study, we applied an unbiased CRISPR/Cas9 in vivo screening approach to identify key enzymes involved in lipid metabolism in T cells in vivo and found mtFAS and *Mecr* to be critical for T cell recruitment and persistence in inflammation. Our results show that MECR regulates CD4^+^ T cell function and oxidative metabolism. Given the role of MECR in mtFAS, it was surprising that naïve *Mecr*-KO T cells were largely unaffected. Nevertheless, *Mecr*-KO cells exhibited significant fitness disadvantages with reduced functionality in IBD. However, it is also interesting to consider the global effects of mutating this pathway, as global knockout of *Mecr* is embryonically lethal in mice and overexpression causes cardiac dysfunction,^56^^,^^57^ but MECR deficiency did not eliminate T cells.

Interestingly, MECR had context-specific effects on CD4^+^ T cell subsets, with Th1 cells having no effect in acute in vitro assays, whereas IFNγ and T-bet were decreased with *Mecr*-KO in chronic in vivo IBD models. In addition, in vitro Th17 cells had reduced IL-17a^+^ and increased FoxP3^+^ T cells. While natural Tregs appeared unaffected, *Mecr*-KO in iTregs led to increased IL-10 production and a significant increase in FoxP3 expression, which may suppress inflammation. This finding was somewhat unexpected, as we hypothesized that *Mecr*-KO would negatively affect Tregs due to their reliance on fatty acid oxidation and OXPHOS for energy, as compared with aerobic glycolysis and fatty acid synthesis in effector T cells.^1^ While differences in proliferation and metabolism were apparent, discrepancies in vitro and in vivo make it challenging to definitively conclude mechanisms by which MECR differentially impacts T cell subsets. In addition, the observation of increased cytokines in our differentiation experiments is consistent with previous studies in which mitochondrial disease patients had an integrated stress response with increased secretion of cytokines.^58^ Therefore, our data support a model in which MECR mediated metabolism broadly supports CD4 T cell proliferation and survival in vivo and lead to context specific effects on differentiation.

MECR-deficient skeletal myoblasts were previously shown to have reduced basal respiration and low spare respiratory capacity. This was in part due to reduced acyl-ACP in complex with LYRM proteins to help form ETC complexes. Consistent with this, ETCs I, II, and IV were almost completely absent in MECR-deficient skeletal myoblasts.^13^ Impaired oxidative metabolism and ETC deficiency were also observed in MECR-deficient T cells along with a severe reduction in lipoic acid on DLAT and DLST. Reduced mitochondrial respiration was consistent with other studies showing that LYRM proteins help stabilize ETC complexes I, II, and IV.^13^^,^^59^ However, depending on mutations in the pathway, lipoic acid synthesis may be retained, as shown by a case study with a patient harboring a mutation in *MCAT*,^59^ confirming that lipoic acid is not the only biologically important product generated by the mtFAS pathway. It was possible that T cells obtained lipoic acid from serum in cell culture media, but previous studies found that supplementing with lipoic acid does not rescue ETC assembly and lipoic acid must be made endogenously to post-translationally modify enzymes as they are synthesized and fold into protein complexes.^10^^,^^13^ In addition, studies have shown little to no difference in the overall cellular lipid composition of *Mecr*-KO cells, with a recent study only showing a difference in the accumulation of ceramides.^15^ Our data support that MECR is needed in T cells to support ETC complex stability and lipoic acid biosynthesis.

Metabolomic analysis of TCA cycle intermediates showed reduced levels, and flux of mitochondrial oxidative metabolism and transcriptomics analysis revealed reduced expression of genes from the OXPHOS pathways in vivo. Total levels of citrate, succinate, fumarate, and malate were each reduced in *Mecr*-KO cells. The contribution of glucose to the TCA intermediate citrate was also reduced, suggesting increased glutaminolysis to replace glucose oxidation. Indeed, glutamate levels were reduced and glutamine levels showed a trend to decrease, but α-ketoglutarate remained high, consistent with glutamine anaplerosis. However, mitochondrial general defects in electron transport capacity may limit this affect, and it has previously been found that in skeletal myoblasts glutamine cycling in the TCA cycle was reduced, but there was no different in glutamine uptake.^13^ Reduced NAD^+^ and ATP suggest metabolic stress and an inability of *Mecr*-KO T cells to produce adequate ATP. Cumulatively, depletion of ETC interacting TCA intermediates in *Mecr*-KO CD4^+^ T cells support ETC dysregulation. Our data also suggest that excess mtROS in MECR-deficient T cells may cause reduced T cell function and that the reduced mitochondrial function could contribute to reduced T cell memory and dysfunction in vivo.

MECR-deficient T cells also had increased levels of labile iron, mitochondrial iron, and CD71. This correlates well with *Mecr*-KO in *Drosophila* and fibroblast MEPAN patient samples also having an accumulation of intracellular iron.^15^ Consistent with elevated iron accumulation, *Mecr*-KO CD4^+^ T cells were more susceptible to ferroptosis, which may contribute the fitness disadvantages observed in vitro and in vivo. However, because mtFAS generates products that contribute to multiple different pathways, we anticipated that we could not fully rescue CD4^+^ T cell function by solely targeting ferroptosis. Regardless, these findings corroborate oxidative metabolomic data, in which ETC complexes I and II, which require Fe-S clusters, are reduced. As iron is essential in T cells and can control mitochondrial function and T cell activation through Fe-S clusters and mtROS,^60^ this is another way in which MECR deficiency may reduce activation and function of T cells.

Interestingly, patients with mitochondrial disease often have immune dysfunction, with recurrent upper respiratory infections that can lead to sepsis, pneumonia, and other health complications such as gastroenteritis and leukopenia.^61^ While we would expect MEPAN patients to exhibit similar immune dysfunction based on our data, no previous case report has evaluated immune function, possibly due to the rarity of the patients and overall complexity of the disease presentation. Our data show that MECR and the mtFAS pathway are vital for the function of effector T cells in vitro and in vivo. This study may provide opportunities to further investigate mtFAS in immune cells and its immunological impact in MEPAN and mitochondrial disease patients.

## Supplementary Material

vkaf034_Supplementary_Data
