# Supplementary material for: Mitochondrial fatty acid synthesis and MECR regulate CD4+ T cell function and oxidative metabolism
Source: J Immunol. 2025 Apr 9;214(5):958–76. doi: 10.1093/jimmun/vkaf034 (PMC12123211; doi:10.1093/jimmun/vkaf034)
Supplement: vkaf034_Supplementary_Data [file vkaf034_supplementary_data.pdf]

## Supplemental Figures and Legends

**Supplemental Table 1: CRISPR/Cas9 Lipid metabolism sgRNA library.** Table shows list of all genes and positive controls in the lipid metabolism library as well as the relevant corresponding lipid metabolism pathway and sgRNA sequences.

**Supplemental Table 1. Lipid metabolism gRNA Library**

| Genes | Pathway | sgRNA 1 | sgRNA 2 | sgRNA 3 | sgRNA 4 |
|---|---|---|---|---|---|
| Acaa1a | Fatty Acid Oxidation | TGTGTGCACTAGGATGACCT | GACACCCTCATCCTGAGACA | CGAGGCCTGCGGGAACCTAG | GGGACATGGACTCCACCCTA |
| Acaa1b | Fatty Acid Oxidation | AACCACTGTCCTGAATGACA | GGCGGGAAGAAATATTCCCA | TGTGTGCACTAGGATAACTT | GGAGCCAGAGAGATTACCTC |
| Acaa2 | Fatty Acid Oxidation | GCCCACGATGACACTATCGA | GCTGAGGTCGTCTTGTGTGG | CGAGGCTGGCTACTTCAATG | CGTCCGTGTTCAAGAAAGAC |
| Acox1 | Fatty Acid Oxidation | CGATCCAGACTTCCAACATG | AATGTCGGATGGCTTGCGGT | CCTCACAGCACTGTATCGAA | TCTCTTCATAACCAAACTTG |
| Acox3 | Fatty Acid Oxidation | CAAGACGGCAACTCATGCGG | GCCTCATGGACAGCCCAGCG | TCAGCACTAGAATCTTCAGG | GCGCATACTTCCGTACCTGG |
| Acsl1 | Fatty Acid Oxidation | GATGTCAGAACCATGTACGA | ATCACCTACATAGTGAACAA | TTCCACCAGATCACTGCCGT | TATGTTTGAGACCGTTGTAG |
| Cpt1a | Fatty Acid Oxidation | CACATTGTCGTGTACCACAG | CATACTGCTGTATCGTCGCA | ACCTTGGACCCAAATTGCAG | ACGTTGGACGAATCGGAACA |
| Cpt1b | Fatty Acid Oxidation | CCTCAACACCGAACACTCGT | CTCGAACATCCACCCGTGGT | TGATGAAGCATATTACCGCA | TAAGACTGGTCTCATCGTCA |
| Cpt1c | Fatty Acid Oxidation | CCACTCGGACAGGAGTATGT | TCATACTGGGCGGAACACAA | CATTTGCCAGCGCTGTAGCG | TGGGAGAGTGAGTCCCTACA |
| Cpt2 | Fatty Acid Oxidation | TCACTGGTCAAATAAGCCAG | TCGGGAAGTCATCTAAGCAG | AAATATTGGGACATATCCAG | TTAAATACATATCAAACCAG |
| Dgat1 | Fatty Acid Oxidation | AGTGGTTTCAGCAATTATCG | AAAGCGCTTTCGTATTCGGG | ATACCCGGGACAAAGACGGG | GCTCACCAATAATCACGCAT |
| Echs1 | Fatty Acid Oxidation | AGTCGGCAAATCGCTAGCAA | GACAGCCAGAAATCCTCCTG | GATGACCGGTTTCTTGACCC | GTGCATTGAGTGCTTTGGGG |
| Ehhadh | Fatty Acid Oxidation | TTCATATGGATGCTTCACGG | CCACATCATGAGGTTACTAG | GTAAACCCATAGAACCCCGC | TCACTATGGCTCTAACCGTA |
| H2-Ke6 | Fatty Acid Oxidation | TGCGATCAGCGTGCGCCTAG | TTCCAAGCGGATGTGTCTCA | CAGGACACAACGACAGATGG | CATTAGTAGCATCATTGGAA |
| Hadh | Fatty Acid Oxidation | CCTTTCAACCAGCACCGATG | AGCAAATCGGTCTTGTCTGG | AAGCATGTGACCGTCATCGG | TGGCCATACAGTAGTATTGG |
| Hadha | Fatty Acid Oxidation | ACACTTTGTGCTTTACACCG | TATTAATTATGGCGTCAAAG | TTAAAGACACCACAGTGACG | AAGGACAATTGAATACCTAG |
| Hadhb | Fatty Acid Oxidation | ATATACAAGTCTTACCTCAG | CTGACAGCAGAAATGGAATG | GCATCGGACCAATATTCCAA | TCAAACCAAGCCATGACCAC |
| Scd1 | Fatty Acid Oxidation | ATGATAAGGAAGATCCGCAG | AGGGGCGCTGCTCACCGAAG | TCTCGTTCATTTCCGGAGGG | GGATGAAGCACATCAGCAGG |
| 1700061G19Rik | Fatty Acid Synthesis | ACAGCTGCAAAAGATTAACC | TCCCGACTTGATCATGAGCG | TGATAGGGATGTTTACGCTG | TATATAACCAGGGTACCACA |
| Acaca | Fatty Acid Synthesis | GCCATTCATTATCACTACGT | AATGCATGCGATCTATCCGT | TTGATTCATAGGTACCGAAG | AAGCCCTTCGAACATACACC |
| Acacb | Fatty Acid Synthesis | AGAACAACGATATCGACACG | CCAGTGTGCCGACCACATGG | GTGGCCGTACATGTCGATGG | TTCATTACGGAACATCTCGT |
| Acat1 | Fatty Acid Synthesis | GTCCCATACGTAATGAGCAG | AGTGCTATAAGAACTCCCAT | AGGCAAGCAACACTGGGCGC | GCCTCTCAAAGTCTTATGTG |
| Acat2 | Fatty Acid Synthesis | CAAACCTGATGCGTTCGCTG | CTGAGAACAGGAGTCAGGAT | TCCTACTCGACAAGCCAGTG | TGCCTTTCACAACTACCACA |
| Acly | Fatty Acid Synthesis | GAGAGAGATTGACCCCGACG | AGAGCGATTCGAGATTACCA | TTGTCACCTGTACACGACGG | GGACGAAAAGCTGAATACCG |
| Acsbg1 | Fatty Acid Synthesis | AGCACTATGGACAACCCGGG | GGACCCTGGTAAACACACTG | CTGCCGAGCCAATGTCATCG | GGATTACCTAGTGTTAGCCA |
| Acsbg2 | Fatty Acid Synthesis | TTCTTGAAGCGTGTACACCA | TCCCGTAGATCTCACCAATG | CCTGACCTTCGCGAACACAA | CACTATCAAGATACTCACGT |
| Acsf3 | Fatty Acid Synthesis | CACAGCGGCTAGGTTACGGT | CATGAATACGGTAATCTGTG | GGCCCACTGTGCTACAACGT | ATATGGCCATCACACCTACA |
| Acsl3 | Fatty Acid Synthesis | TGATCACAACGTACCCACGT | ACATCATTGCTTCTATAACG | TAATGATATGCCGCAGACGT | TTATCAAGTGTATCGCAGCC |
| Acsl4 | Fatty Acid Synthesis | GTCCAGGGATACGTTCACAC | GCCCATATCCCTGACCAATG | CAATAGAGCAGAGTACCCTG | GGAACAGCGGCCATAAGTGT |
| Acsl5 | Fatty Acid Synthesis | CTCTGCTCGATCAGACACCT | TAGACCCTGTTAAGGAGTCG | CGTCATCAAAAGGGTCCATG | GTACCCAGCGTGTCATACAG |
| Acsl6 | Fatty Acid Synthesis | AGAGAATCATCATGCCCCCG | CTGCTACACGTATTCCATGG | TGGCAGTAGACAACAGACTG | GCCCGGCGATCTGTGATTGG |
| Acss2 | Fatty Acid Synthesis | GCTGGGAACCTACTACCCGG | CAGAACGCCGGTGCAGCTCG | AAGGGAAAATATTCACTGAG | GCATTGTGGTCAAACATCTG |
| Dgat2 | Fatty Acid Synthesis | GATCTGCCCTGTCACGCGAG | CTGGCTCAACAGATCTAAGG | AAGGCCCTATTTGGCTACGT | GTCTCGGAAGTAGCGCCACA |
| Fasn | Fatty Acid Synthesis | CTACCAGGCCATCCGTAGTG | TGTCTCCGAAAAGAGCCGGG | TTGGTGGAGCCAATTAACAG | ACTGGCAATCTGATTGTGAG |
| Mlxipl | Fatty Acid Synthesis | CCAGGCTCAAGCACTCGAAG | TGATGCGCGAATACCACAAG | TTCCCTCAGATGTGCAACGG | TGAGCAGCTGTGTAAGACAG |
| Mttp | Fatty Acid Synthesis | TGAGCGGTCTGGATTTACAA | TGATCAAGTGATCCAAGTCA | GATATACCACCAGAATCGTA | ATCCTTTGCAGACACGCTCG |
| Olah | Fatty Acid Synthesis | AAAACCAGAACTTACGTGAG | TGCATGCTGTAAGACTGGCT | TTACAAGATCTAAATACCTG | ATTAATCTTTCGGCCCCACT |
| Cbr4 | mtFAS | CATACCGCCGAGCTCGCCGG | GGAGGGTCTATTGTTAATGT | TGGTGGCACTGTATGCAGAC | AATTTCTTGGTAAATGCAGC |
| Mcat | mtFAS | TATCTGGTTAGGTCTGTATG | GGTCATCTCAGGACACCTTG | ATGATGTAGCTTCTCCACGG | AGGAGAAGTTGGACTGACGC |
| Mecr | mtFAS | CGTGGCGGTACCAAGCCTCG | AAGGATCTGACGTCCACGTG | ATCCAGAATGCATCCAACAG | AGCACTGATTGGAATCCCTA |
| Oxsm | mtFAS | GACAACTCGTCTGCGCAACA | GTAGCTGCTTATGTACCAAG | AGCATTCGCTACAAACTCAA | CCATCACATCTGCGTCACCA |
| Nr1h3 | Steroid Biosynthesis | AAAGCAACCCCAGTTGACTG | CAAGTACCGTGACGCGCAGG | AATCTCCTGCACGGACACGA | TTCCGCCGCAGTGTCATCAA |
| Pparg | Steroid Biosynthesis | AATGCTGGAGAAATCAACTG | AGAACCTTCTAACTCCCTCA | GCACCCTTGAAAAATTCGGA | CTGCCTATGAGCACTTCACA |
| Srebf1 | Steroid Biosynthesis | CAGGCTCGAGTAACCCAGCA | AATGCCCCAGCCGAAAAGCG | TCTGCCTACAGAATCACTGA | ACTGCAGCCACACTTCATCA |
| Srebf2 | Steroid Biosynthesis | GATGATCCTCGTCTGCGGAG | AGCGACCGTCTGTACCGTGG | ACTCCAGTGACAGTACACTG | CCTTACTGGCACTTGAAGGG |
| Rheb | Positive Control | AACAAACTGAATTGTCAATG | CCATATCCAACAACTTGCCA | TTCAGCTTGTAGACACAGCG | TCATAGGATACCTATTATGT |
| Tsc2 | Positive Control | TGAACCACATGGCTATGACG | CACAGGGTGATAATGAACAG | CAGCTCCAAAGACCCTTGAG | CTGATCCTAGCACACATGTG |
| NTC_BRDN0000737434 | Negative Control | AAACTCCCGTGTCAACCGAT | NA | NA | NA |
| NTC_BRDN0000737467 | Negative Control | AAACCTAGCGTAGATTCGGC | NA | NA | NA |
| NTC_BRDN0000737505 | Negative Control | AAAAAGTCCGCGATTACGTC | NA | NA | NA |
| NTC_BRDN0000737609 | Negative Control | AAACTCATACGTAGCGAATC | NA | NA | NA |
| NTC_BRDN0000737637 | Negative Control | AAAACGTAATTATACCGAGC | NA | NA | NA |
| NTC_BRDN0000737693 | Negative Control | AAAACGGCTCGATCGGTGAT | NA | NA | NA |
| NTC_BRDN0000737801 | Negative Control | AAACCCCCGCGCGGAGCGTC | NA | NA | NA |
| NTC_BRDN0000737848 | Negative Control | AAACGAGGCTGTTCGTACAC | NA | NA | NA |
| NTC_BRDN0000738185 | Negative Control | AAAATTGCACCTTCCCGGCC | NA | NA | NA |
| NTC_BRDN0000738254 | Negative Control | AAAGACGTGCATTCAGCGAG | NA | NA | NA |

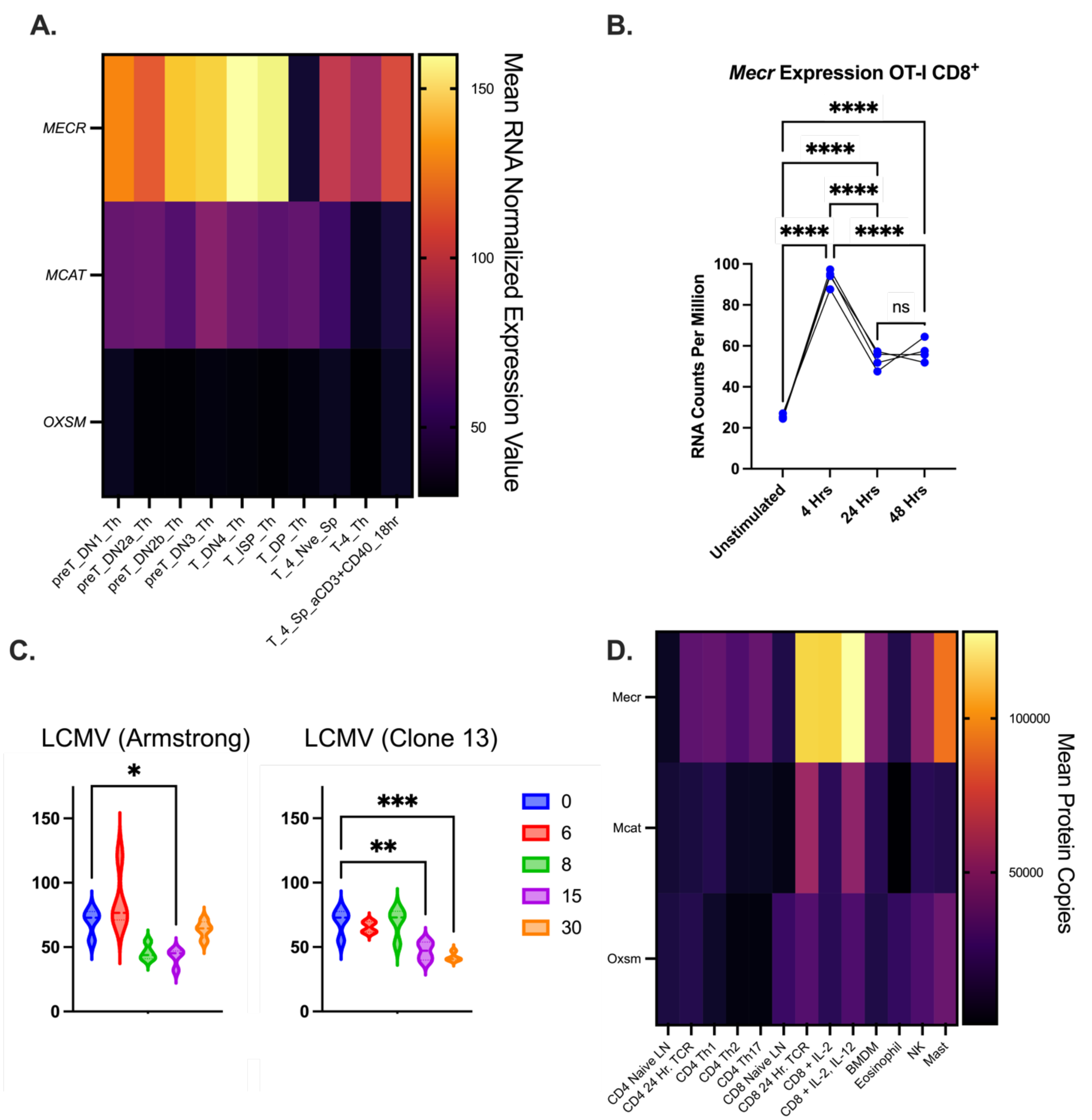


**Supplemental Figure 1: *Mecr* expression changes upon T cell activation. a)** Mean normalized expression values of mtFAS human genes from the Immunological Genome Project database (ImmGen) (48). Labels are the following: preT_DN1_Th- Thymic preT Double Negative 1, preT_DN2a_Th- Thymic preT Double negative 2a, preT_DN2b_Th- Thymic preT Double Negative 2b, PreT_DN3_Th- Thymic preT Double Negative 3, T_DN4_Th- Thymic Double Negative 4, T_ISP-Th-Intermediate single positive thymocytes, T_DP_Th- Thymic double

positive, T_4_Nve_Sp- Splenic Naïve CD4, T-4_Th-CD4+ Thymocytes, T_4_Sp__aCD3_CD40_18hr- Splenic activated $CD4^+$ T cells, aCD3+CD40 18 Hr. **b)** Quantified *Mecr* expression time course in activated antigen-specific murine OT-1 $CD8^+$ T cell RNAseq from GSE232241 (17). **c)** Expression levels of *Mecr* mRNA in $CD8^+$ T cells that specifically recognize H2-Db GP33 LCMV antigen during infection. $CD8^+$ T cells were isolated from mice infected with acute (LCMV-Arm) or chronic (LCMV-C13) variants of the virus at different days post infection. The data are reanalyzed from GSE41870 (49) **d)** Mean human protein copies of mtFAS genes from the Immunological Proteomic Resource (ImmPRes). Panel (b) each data point represents a biological replicate and error bars show standard deviation. Panel (b) statistical significance performed by one-way ANOVA with Tukey's multiple comparison post-hoc test. Panel (c) statistical significance performed by one-way ANOVA with Dunnett's multiple comparison post-hoc test. (* $p<0.05$, ** $p<0.01$, *** $p<0.001$, **** $p<0.0001$).

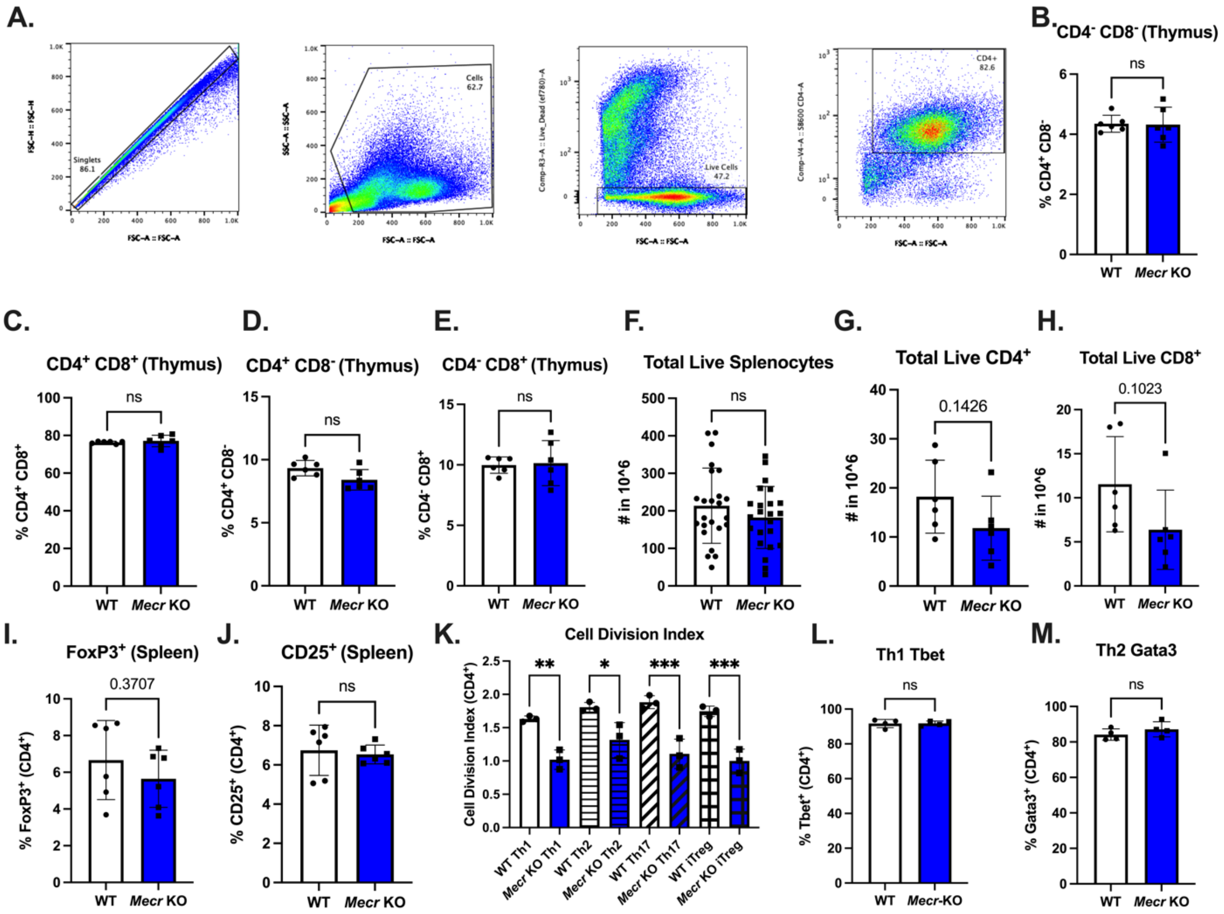


**Supplemental Figure 2: *Mecr*$^{fl/fl}$; *Cd4*$^{cre}$ mice display no difference in thymocytes and T regulatory cells *ex vivo*. a)** Gating scheme for all flow cytometry experiments. **b-e)** CD4/CD8 thymocytes of live cells. **f)** Total live splenocytes of *Mecr*$^{fl/fl}$; *Cd4*$^{cre}$ mice**. g)** Number of live CD4$^+$ T cells. **h)** Number of live CD8$^+$ T cells. **i, j)** Quantification of T regulatory cells ex vivo of FoxP3$^+$ CD4$^+$ T cells and CD25$^+$ T cells. **k)** Cell division index from CTV in T cell subsets. **l)** Percentage of Th1 Tbet$^+$ skewed cells. **m)** Percentage of Gata3$^+$ Th2-skewed cells. All panels were conducted by flow cytometry. Panels (b-j) show results from two pooled independent experiments. Panel (k) shows results from one independent experiment. Panels (l, m) show representative results from six independent experiments. Each data point represents a biological replicate and error bars show standard deviation. Panels (a-j, l, m) statistical analysis significance performed by unpaired

t tests. Panel (k) statistical analysis for significance performed by two-way ANOVA with Tukey post-hoc test (* $p<0.05$, ** $p<0.01$, *** $p<0.001$, **** $p<0.0001$).

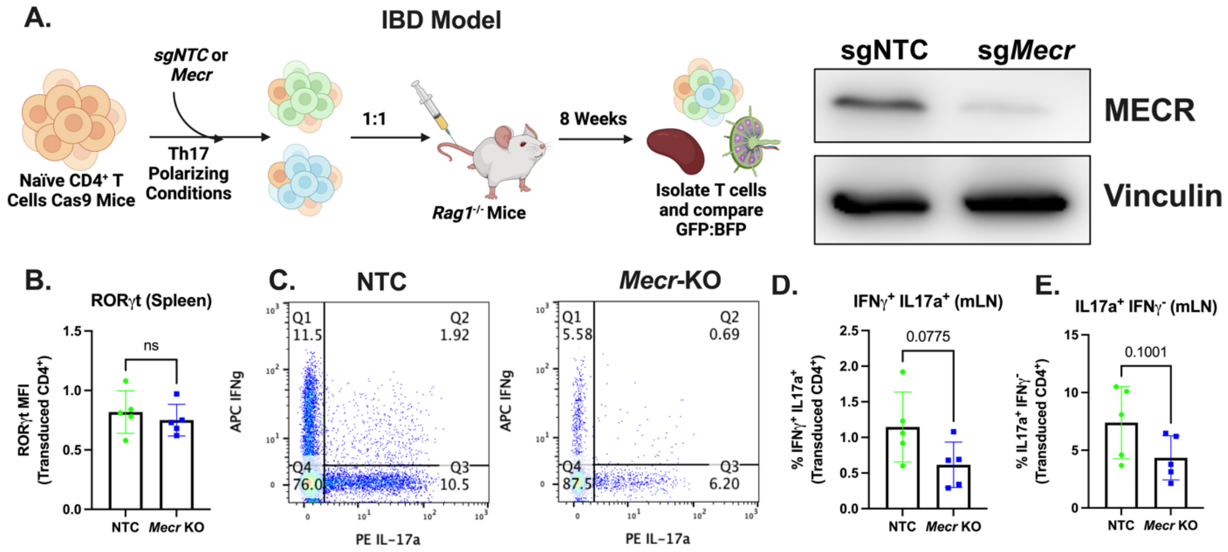


**Supplemental Figure 3: *Mecr*-KO *in vivo* causes reduced IFNγ/ IL-17a and IL-2 in an *in vivo* model of IBD using CRISPR/Cas9. a)** Experimental scheme of *in vivo* 1:1 IBD CRISPR/Cas9 protocol. **b)** RORγt expression pre-adoptive transfer. **c,d)** Percentage of IFNγ$^+$ and IL-17a$^+$ post-disease. **e)** Percentage of IL17a$^+$ IFNγ$^-$ post-disease. Panels (b-e) show representative results from three independent experiments. Each data point represents a biological replicate and error bars show standard deviation. Statistical significance performed by unpaired t tests. (* p<0.05, ** p<0.01, *** p<0.001, **** p<0.0001).
